# Ancient oral microbiomes support gradual Neolithic dietary shifts towards agriculture

**DOI:** 10.1038/s41467-022-34416-0

**Published:** 2022-11-22

**Authors:** Andrea Quagliariello, Alessandra Modi, Gabriel Innocenti, Valentina Zaro, Cecilia Conati Barbaro, Annamaria Ronchitelli, Francesco Boschin, Claudio Cavazzuti, Elena Dellù, Francesca Radina, Alessandra Sperduti, Luca Bondioli, Stefano Ricci, Miriam Lognoli, Maria Giovanna Belcastro, Valentina Mariotti, David Caramelli, Marta Mariotti Lippi, Emanuela Cristiani, Maria Elena Martino, Italo Maria Muntoni, Martina Lari

**Affiliations:** 1 Department of Comparative Biomedicine and Food Science, University of Padua, Legnaro, 35020 Italy; 2 Department of Biology, Laboratory of Molecular Anthropology and Paleogenetics, University of Florence, Florence, 50122 Italy; 3 Dipartimento di Scienze dell’Antichita, “Sapienza” University of Rome, Rome, 00185 Italy; 4 Dipartimento di Scienze Fisiche, della Terra e dell’Ambiente, U.R. Preistoria e Antropologia, University of Siena, Siena, 53100 Italy; 5 Dipartimento di Storia Culture Civiltà, University of Bologna, Bologna, 40126 Italy; 6 Soprintendenza ABAP per la Città Metropolitana di Bari, Bari, 70121 Italy; 7 Sezione di Bioarcheologia - Museo delle Civiltà, Roma, 00144 Italy; 8 Dipartimento Asia, Africa e Mediterraneo, “L’Orientale” University of Neaples, Neaples, Italy; 9 Dipartimento dei Beni Culturali, University of Padua, Padova, 35139 Italy; 10 Department of Biology, Laboratory of Palynology, University of Florence, Florence, 50121 Italy; 11 Department of Biological, Geological and Environmental Sciences, University of Bologna, Bologna, 40126 Italy; 12 DANTE - Diet and ANcient TEchnology laboratory, Department of Maxillo-Facial Sciences, “Sapienza” University of Rome, Rome, 00161 Italy; 13 Soprintendenza Archeologia, Belle Arti e Paesaggio per le Province di Barletta - Andria - Trani e Foggia, Foggia, 71121 Italy

**Keywords:** Microbiome, Archaeology

## Abstract

The human microbiome has recently become a valuable source of information about host life and health. To date little is known about how it may have evolved during key phases along our history, such as the Neolithic transition towards agriculture. Here, we shed light on the evolution experienced by the oral microbiome during this transition, comparing Palaeolithic hunter-gatherers with Neolithic and Copper Age farmers that populated a same restricted area in Italy. We integrate the analysis of 76 dental calculus oral microbiomes with the dietary information derived from the identification of embedded plant remains. We detect a stronger deviation from the hunter-gatherer microbiome composition in the last part of the Neolithic, while to a lesser extent in the early phases of the transition. Our findings demonstrate that the introduction of agriculture affected host microbiome, supporting the hypothesis of a gradual transition within the investigated populations.

## Abstract (web summary)

Here, the authors compare 76 dental calculus oral microbiomes from Palaeolithic hunter-gatherers with Neolithic and Copper Age farmers living in the same region of Italy. Integrating these data with archaeological data and dietary information, they find evidence of a gradual transition to agriculture.

## Introduction

In the last decade, several studies have identified the microbiome as a type of interface between humans and their surrounding environment. The human microbiome is able to influence host health through several mechanisms^1,2^, and it is in turn shaped by variables that are related to host life conditions (e.g. lifestyle, environment), more so than by host genetics^3,4^. Since the beginning of metagenomic analyses of the human microbiome, the existence of huge degrees of variability within gut microbiome composition among different populations across the world has been highlighted^5,6^. Nonetheless, how these microbial communities arose during human evolution and co-evolved with their hosts remain open questions.

Ancient DNA (aDNA) studies may potentially enable us to explain how these complex microbial communities evolved over time to reach their current composition, and moreover, they may provide substantial information regarding past societies, cultures and life conditions^7,8^. Researchers are now considering the study of the ancient microbiome from coprolites (i.e., fossilised faecal remains) and dental calculus as tools that could considerably contribute to revealing the evolution of human-associated microbial communities^9,10^.

One of the outstanding questions regarding the evolution of the human microbiome is related to how the transition to agriculture during the Neolithic period shaped oral microbiome composition and biodiversity. Analyses conducted on modern populations have detected changes in oral microbial ecology when comparing traditional or hunter-gatherer societies with urban communities^11–14^. However, recent studies that have focused on the transition to agriculture over our past have shown controversial results. For instance, preliminary ancient oral microbiome data suggested a shift in bacterial species abundance linked to the Neolithic transition, which was not replicable in a more recent study on oral microbiome evolution^8,15^. Additional research in Southern Europe found little variation associated with the transition, identifying mainly geographical patterns of bacterial genome variation^16^. To date, studies on the ancient Neolithic microbiome have mostly relied on small groups of samples distributed over a broad geographical area characterised by distinct ecological conditions and subsistence strategies, thus introducing possible geographical and dietary bias. Indeed, as observed for different microbiome sources, the microbial communities that inhabit dental plaque appear to be influenced by diet, ecology and living conditions^17–19^. Moreover, archaeological evidence suggests that the Neolithic transition was not a monolithic event, but differences occurred in the adopted subsistence strategies across coeval communities^20–22^. This evidence may account for why, geographic patterns of variation are mainly detected rather than cultural changes^16^, when comparing different Neolithic communities characterised by small sample sizes and different ecological conditions, thus leaving this question open. Accordingly, paying attention to both the archaeological background and the ecological context become fundamental for contextualising the influence of the agriculture transition on the oral microbiome.

In Italy, the transition to agriculture started around 6,200 BC in the southeast of the peninsula, in a region currently known as Apulia, where populations from the Levant reached this land by following migratory routes across the Mediterranean Sea^23^. Here, the shift from hunter-gatherer to agrarian subsistence was documented as the first for Italy, and as one of the most ancient in Western Europe^23^. The first phases were characterised by a few groups of families, organised in small villages with a typical type of ceramic production called “Impressed Ware”^23^. Over the next two millennia, these communities spread northward, developing more complex social structures, and also adapting their agrarian subsistence system in response to local resources and to environmental conditions^24^. In the last decade, this region has been the object of multiple palaeodietary and palaeoecological investigations aimed at reconstructing the dynamics of Neolithisation, thus representing a perfect case study for investigating the relation between the host and the microbiome at the time of transition^24–26^. Overall, the Neolithic period in this area is divided into four main cultural phases: Early (corresp. Impressed Ware culture), Middle (corresp. Passo di Corvo culture), Final Middle (corresp. Serra d’Alto culture) and Late Neolithic (corresp. Diana culture)^23^.

Thus, the aim of the present study is to address questions related to the effects of Neolithic dietary change on the oral microbiome, integrating dietary, cultural and environmental information. We propose an alternative approach to investigating the evolution of the microbiome composition in relation to agricultural transition, minimising possible geographical bias and focusing exclusively on Central-South Italy as a case study (particularly on the Apulia region) and considering the cultural and dietary context of each community. We first analyse nine upper-Palaeolithic hunter-gather samples from Paglicci Cave (31,000–11,000 BC), both to further describe the pre-agricultural microbiome, and to obtain a regional reference to compare with the Neolithic specificities. To date, no Mesolithic individuals have been detected in this area. To better address the changes related to the transition, we focus on a set of 67 samples from the Neolithic (6200–4000 BC) to the Copper Age periods (3500–2200 BC), crossing all four phases that constituted the Neolithic period in this area, and corresponding to distinct cultural backgrounds. Moreover, to expand the informative content of ancient dental calculus, we applied an integrated approach coupling metagenomic data with microscopic analysis to obtain dietary information regarding the plants consumed. We identify two previously undescribed shifts in the microbiome composition of the investigated populations. The first modification occurred between local hunter-gatherer communities and Neolithic farmers, while a second unexpected major change was identified in the last part of the Neolithic period. Some of the taxonomic and functional changes were probably driven by changes in subsistence strategies.

## Results

### Validation of the ancient microbiome profile and aDNA authentication

**Fig. 1:**
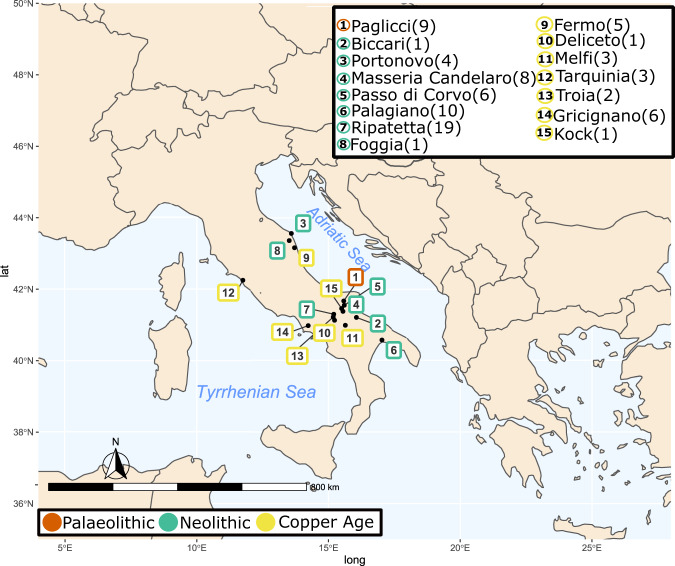
Samples distribution. Sampled archaeological site distribution across the Central-South Italian peninsula. The number of samples per site are presented in brackets. Source data are provided as Source data file. Map was generated using package rnaturaleartch (v 0.1.0) on R software.

We collected 79 samples from Central-South Italy (Fig. 1), considering upper-Palaeolithic (PA) hunter-gatherer, post-Neolithic Copper Age (CA) samples, and crossing all of the different Neolithic phases (Supplementary Data 1): Early Neolithic (EN), Middle Neolithic (MN), the final part of the Middle Neolithic (FMN) and Late Neolithic (LN). Seventy-six (76) of the collected samples were investigated for their metagenomic profiles. A mean of 28 million reads per sample were produced (Supplementary Data 2). Of these, a mean of 12.94% reads could be assigned to Bacteria and Archaea, with only a small portion to Eukaryote (a mean of 0.01%). The obtained ancient microbiome profiles clustered together with other ancient published oral microbiomes and modern oral samples (Supplementary Data 3 and Supplementary Fig. 1). To confirm the robustness of our results, we corroborated them through Sourcetracker analysis, where the majority of the ancient samples (71) showed a strong adherence to the oral microbiome profile (>90%) (Supplementary Fig. 2). Only a few samples showed a contaminated profile, and were thus excluded from further analysis.

We selected the most abundant microbial species that showed a relative abundance greater than 0.02% in the whole dataset. This threshold enabled us to obtain a total of 49 species, avoiding low-abundance species and singletons. The obtained species were consequently validated for their deamination profiles (Supplementary Data 4 and Supplementary Fig. 3). A close deamination profile among all of the samples was detected, without any significant differences being found to occur over time (see Supplementary Discussion and Supplementary Figs. 4 and 5 for more taphonomic information).

### Transition elements in human oral microbiome from Palaeolithic to Copper Age

**Fig. 2:**
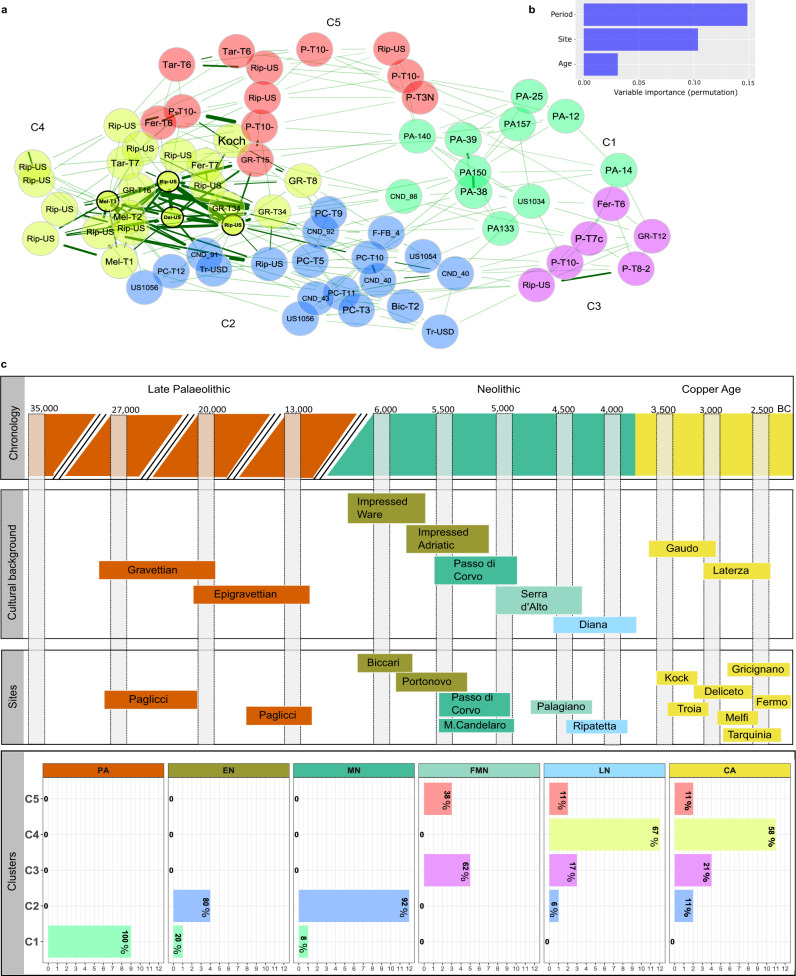
Metagenomic analysis. **a** Network analysis among samples. The edge thickness is based on their weights, which indicate non-negative similarities among samples, while edges length represent nodes distance based on Aitchison distance. Node colour is defined by the cluster identified by hierarchical cluster analysis, and the black border colouration around the node identifies the hubs. **b** Random forest classification analysis results, highlighting the importance of period and geography (site) as the main variables forming the basis of the cluster differences, while the age of death (Age) is not discerning. **c** Correspondence of the investigated time (i.e., chronology), with the cultural background (second block), the selected archaeological site (third block) and the results from the cluster analysis (C1-C5) reported in panel **a**. In the “Cultural background” block, each Neolithic culture is defined by a different colour, which highlights its association to the archaeological sites (third block) and to the specific Neolithic phase reported in the headings of the “Clusters” block (i.e., EN, MD, FMN, LN). In the last block (i.e., called “Clusters”), sample numbers are reported on the x-axis and cluster annotation on the y-axis. Each bars’ colour is associated to clusters ‘colours reported in panel **a**. Within each bar, the percentage of samples falling within a specific cluster is reported in bold. Here, following cluster rows, we can appreciate that PA samples fall together within the first cluster (C1), while EN and MN in the C2. LN and CA are particularly present in C4, while FMN is divided between C3 and C5. Source data are provided as Source data file.

We performed a network analysis to explore sample similarities, detecting five different clusters (C1-5) (Fig. 2a). Cluster subdivision was validated using PERMANOVA and the ANOSIM test on a PCoA representation, confirming that it was genuinely influenced by microbiome composition and not related to other confounding factors (i.e., extraction or library batch, or read counts) (Supplementary Figs. 6,  7 and Supplementary Data 5a and b). According to random forest classification, the time period was identified as the primary factor driving these cluster distinctions, with the geographical location being a secondary factor (Fig. 2b). Thus, we can suppose a mixing effect of time and geography, with the chorological period having a slightly heavier influence than geography. Actually, the association analysis between the clusters and the period (Fig. 2c and Supplementary Fig. 8) indicated that the first cluster (C1) was significantly associated with the PA period, while the second (C2) was primarily associated to Early (EN) and Middle Neolithic (MN) samples. The site of Palagiano, which belongs to the end of the Middle Neolithic (FMN), was associated to both the C3 and C5, thus suggesting the possibility of a further sub-structure within the same site. It is noteworthy that C3 is mostly constituted from samples belonging to the same tomb (P.T.10.x), while C5 is formed from samples belonging to different burials found in Palagiano. Finally, C4 is instead significantly associated with the Late Neolithic (LN) and Copper Age (CA) periods, regardless of geography.

**Fig. 3:**
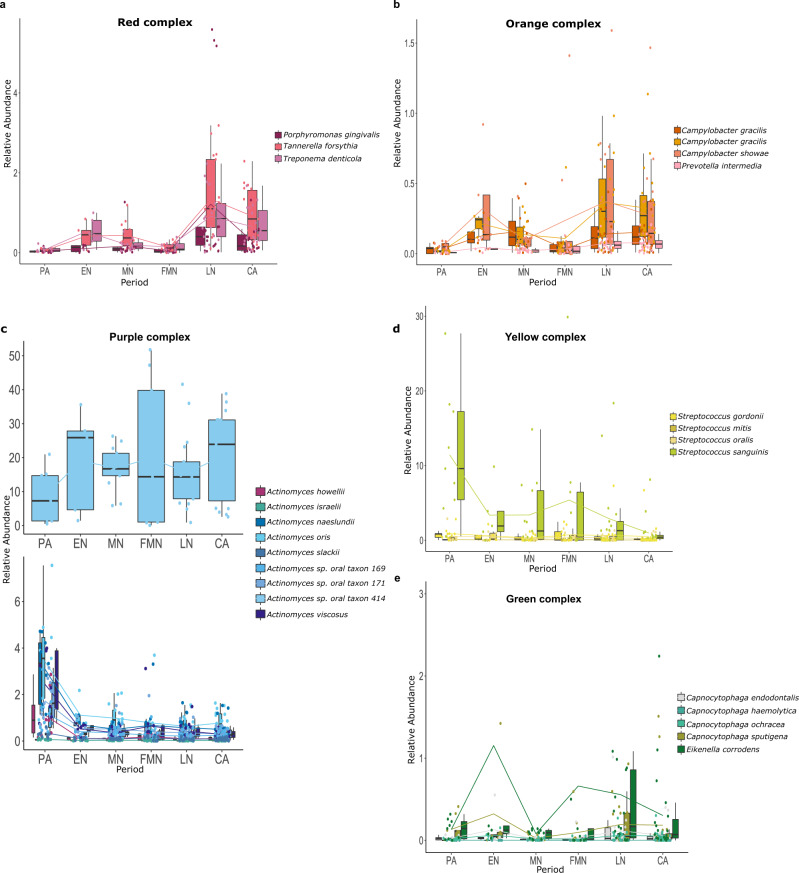
Significant species as identified from DESEq2 analysis and organised in oral microbiome complexes. **a** Red complex; **b** orange complex; **c** purple complex; **d** yellow complex; **e** green complex. Others significant oral species are reported in Supplementary Fig. 10. Samples (*n* = 71) are represented by dots and are coloured in accordance with species. Boxplots are defined as follow: minimum, maximum and centre values represent the 25, 75 and 50% quantile, respectively; median is represented by 50% quantile; upper and lower whiskers are the maximum/minimum values of data which are within the 1.5 interquartile range over 75th or under 25th percentile, respectively. Source data are provided as Source data file.

This evidence suggests the existence of a specific microbiome profile associated with the PA hunter-gatherer samples, and moreover, that some differences are detectable even within the same Neolithic period. Particularly, samples that belong to the first centuries of the Neolithic transition in this area show a highly similar microbiome composition among them, and an evident distinction from those of samples corresponding to the LN and CA periods. Forty-two (42) species were identified from DESeq2 analysis as being differently distributed among the clusters, with some of them falling within well-known ecological complexes for the oral microbiome^27^ (Fig. 3a–e, Supplementary Data 6). Most of the species that belong to the same complex exhibit a similar trend, as expected for being metabolically interdependent and ecologically associated^27^. Among the species that characterise these complexes, we can observe two main taxonomic trends: one for species that are poorly represented in PA samples, but that start to increase with the beginning of the Neolithic transition; and a second trend, whereby species that are more prevalent within the PA samples tend to slowly decrease over time, reaching their lowest levels in LN and CA. Significant species belonging to the red (*Porphyromonas gingivalis*, *Tannerella forsythia* and *Treponema denticola*), orange (*Campylobacter gracilis*, *Campylobacter rectus*, *Campylobacter showae* and *Prevotella intermedia*) and green (*Capnocytophaga endotelialis*, *Capnocytophaga haemolytica*, *Capnocytophaga ochracea*, *Capnocytophaga sputigena* and *Eikenella corrodens*) complexes follow the first trend; thus, they are weakly present in the PA samples, and then they gradually become more heavily represented at the beginning of the Neolithic period, but only at the end of this period do they reach the highest abundance. *Pophyromonas gingivalis* was also positively associated with C4 from MaAsLin analysis, and thus to the LN/CA periods, while *Campylobacter gracilis* was associated with both C2 and C4 (Supplementary Figure 9 and Supplementary Data 7).

Taxa from the purple (*Actinomyces* spp.) and yellow (*Streptococcus gordonii*, *Streptococcus mitis, Streptococcus oralis* and *Streptococcus sanguinis*) complexes followed the second trend, being highly present during the PA period and decreasing their abundance across time. The only exception in this sense from the purple complex was *Actinomyces* sp. *oral taxon 414* (Fig. 3c, upper plot). In addition, also in this case, the MaAsLin result confirmed a strong negative association for all of the cited *Streptococcus* spp. with almost all the Neolithic and CA clusters (Supplementary Fig. 9), while a positive association of *Actinomyces naeslundii* and *Actinomyces slackii* was found with the PA period.

Other members of the oral microbiome significantly changed their abundance across time, such as *Olsenella* sp. *oral taxon 807*, *Parvimonas micra*, *Desulfomicrobium orale* and *Ottowia* sp. *oral taxon 894*, which seem to be widespread among all of the Neolithic samples, while *Streptococcus* sp. *oral taxon 061, Abiotrophia defectica* and *Klebsiella pneumonieae* were enriched within the PA dataset (Supplementary Fig. 10). *Olsenella* sp. *oral taxon 807* demonstrated a strong connection with the Neolithic transition, since MaAsLin highlighted a positive association with both C2 and C4, and thus with the two major Neolithic clusters, while *Desulfomicrobium orale* was associated specifically with the C4 cluster.

Samples from the first phases of the Neolithic period (EN and MN), despite originating from different archaeological sites, showed quite a homogenous taxonomic profile and a microbial composition that appeared to be in an intermediate position between the PA and LN/CA periods. We can thus hypothesise that the first phase of the Neolithic constitutes a type of “transitional state” between the two extremes of the PA and LN/CA microbiome compositions. They are characterised by elements that are in common with LN and CA, and at the same time, they do not seem to be so distant from the compositions of the hunter-gatherer samples.

### Functional analysis

In addition to the taxonomic composition, we also characterised the microbial functions in order to corroborate previous analyses, and to test how these changes may be reflected in a microbial genetic functional package. We detected several KEGG orthologs as being specific to each of the taxonomic-based clusters (Supplementary Fig. 11).

**Fig. 4:**
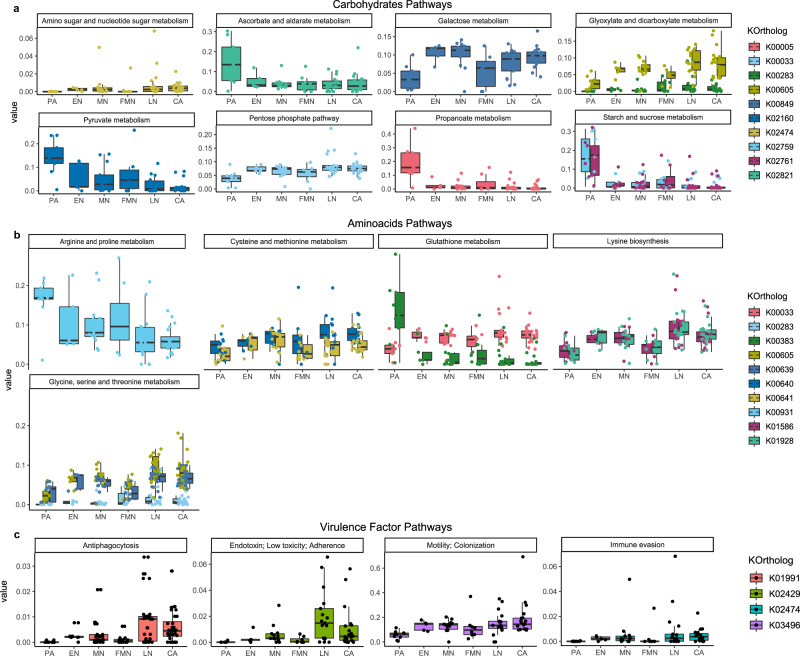
Results from HUMAnN2 analysis. Panels **a** and **b** report differences in carbohydrate and amino acids metabolism from the PA to CA period. Panel **c** represents an increase in several virulence factors over time based on the Chen et al.^91^ classification. Samples (*n* = 71), divided by Periods, are reported on the x-axis, and the relative abundance on the y-axis. Boxplots are defined as follow: minimum, maximum and centre values represent the 25, 75 and 50% quantile, respectively; median is represented by 50% quantile; upper and lower whiskers are the maximum/minimum values of data which are within the 1.5 interquartile range over 75th or under 25th percentile, respectively. Source data are provided as Source data file.

KEGG orthologs that are related to carbohydrate metabolism (e.g. pyruvate, propanoate, ascorbate, amino sugar, and starch metabolism) are found to be particularly enriched in PA samples (Fig. 4a). These traits were recently found to be associated mainly with *Streptococcus* presence, which is higher within this group of samples, and with its ability to bind host amylase proteins^8,28^. However, the Neolithic samples are all enriched in other distinctive features within carbohydrate metabolism, such as galactose metabolism and glyoxylate/dicarboxylate metabolism, as well as augmentation in glycan pathways (Supplementary Fig. 12A).

Multiple significant orthologs belonging to the amino acid pathways seem to increase over time, such as lysine biosynthesis, and glycine and cysteine biosynthesis, which reach a higher abundance in the LN and CA samples (Fig. 4b).

Even among vitamin metabolism, we found significant pathways that changed across time, especially for the B-vitamins (Supplementary Fig. 12B). Differences in enzymes that are part of the biosynthetic pathway to cobalamin (vitamin B12) in bacteria are present in both the PA and in the Neolithic samples. Cobalt-precorrin-6B (K02191) and L-threonine kinase (K16651) are mainly represented within the PA samples, with a tendency to decrease over time, while percorrin-6Y C5,15 methyltransferase (K00595) and adenosylcobinamide-GDP ribazoletransferase (K02233) increase across the Neolithic phases. Neolithic samples, especially LN and CA, are enriched in the one-carbon pool by folate metabolism, as well as in lipoic acid metabolism; lipoic acid is another B-complex vitamin that seems to affect bacterial virulence and pathogenicity^29^.

Interestingly, we detected a general increment in virulence factor among our Neolithic cohort (Fig. 4c). KEEG orthologs involved in bacterial motility, immune evasion, antiphagocytosis and endotoxins gradually increased their relative abundances over time, reaching their highest levels in LN and CA. The species that contributed to the increased levels of these virulence factors were *P.gingivalis*, *T. forsythia*, *P. intermedia*, *Capnocytophaga ochracea* and *Fusobacterium nucleatum*.

Overall, as observed in the taxonomic analysis, subjects that belonged to both EN and MN had a very close functional composition (as highlighted by exploratory cluster analysis), which was a kind of intermediate position between the two more different states observed in the PA and LN-CA samples.

### Microscopic analysis from dental calculus

The nine dental calculus samples belonging to the upper-Palaeolithic (PA) samples yielded mainly starch grains, with respect to other detected types of microremains, for a total amount of 215 single starch grains (Supplementary Discussion and Supplementary Data 8). The starch grains were mainly associated with Poaceae, and particularly with wild Triticeae and *Avena* cf. *barbata* (oat, cf. slender wild oat). Finally, particular starch grains were morphologically identified as those of the rhizome of *Nuphar lutea* (yellow water lily). Water lily starch grains (not yellow water lily) have been found also in Neanderthal dental calculus^30^.

For the Neolithic and Copper Age periods, 18 samples were analysed (Supplementary Data 8). Overall, 25 starch grains belonging to two morphotypes were retrieved from the samples. Morphotype I grains showed diagnostic features for the type A grain of the *Triticeae* tribe species. Among the numerous phytoliths, 16 elongate dendritic epidermal were recovered. These types of phytoliths in dental calculus are normally considered to be a direct evidence of cereal consumption, as they are produced in the inflorescence bracts of wheat and barley species^31^. Interestingly, numerous diatoms of the genus Nitzschia were recovered, which included many freshwater and marine species, which are generally pollution-tolerant^32^. Finally, it is worth mentioning the recovery of fungal spores from seven individuals from the Neolithic and CA periods. Some of the spores were identified as belonging to Glomus, a large fungal genus that is associated with plant roots^33^, while several yeast spores were identified in two Neolithic samples belonging to different sites, probably suggesting the consumption of fermented foods.

For more details regarding micro-debris analysis, please refer to the Supplementary Discussion.

### Reconstruction and phylogeny of *Olsenella* sp. oral taxon 807

**Fig. 5:**
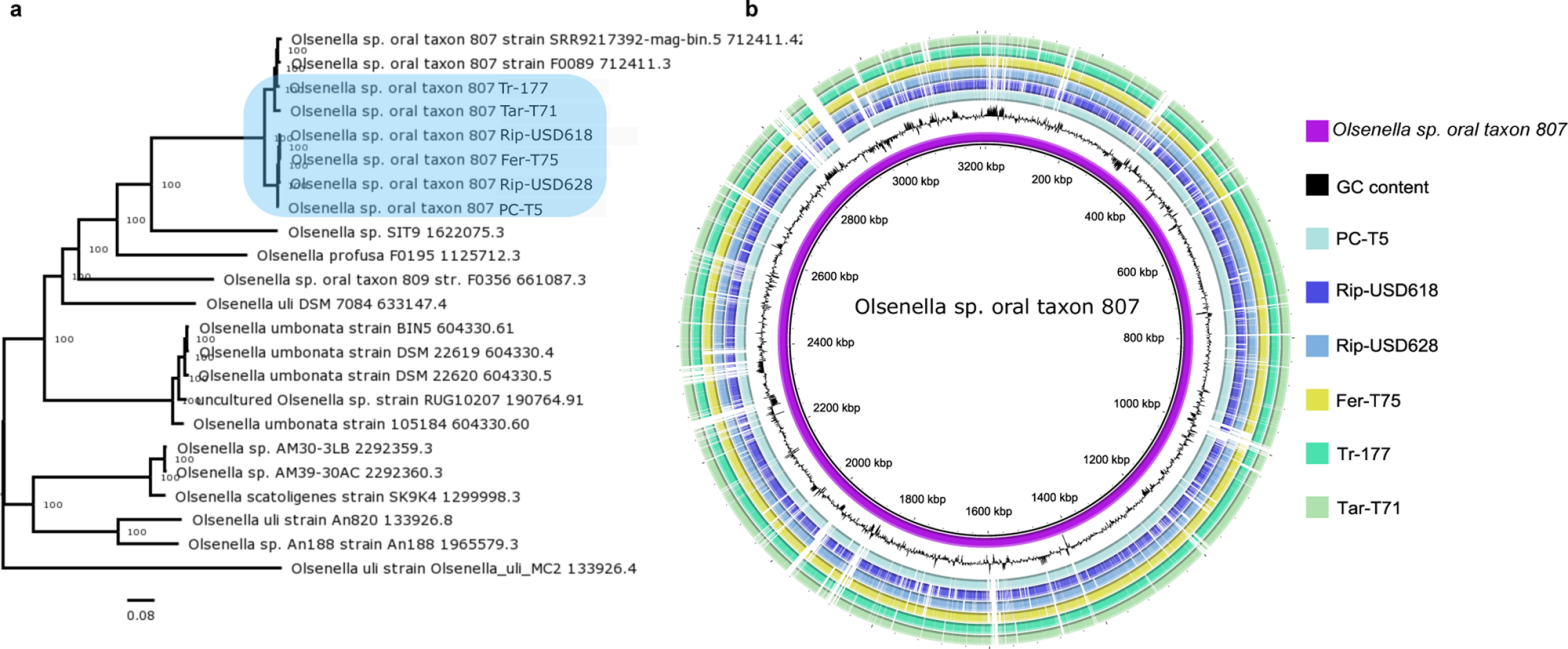
Ancient *Olsenella* sp. oral taxon 807 reconstruction. Panel **a** reports the phylogenetic analysis of the six reconstructed ancient genomes (light-blue box), together with 17 modern Olsenella sp. references, as performed by PATRIC^100^. In Panel **b**, it is possible to observe the alignment analysis conducted with BRIG^99^. Purple circle indicates the *Olsenella* sp. *oral taxon 807* reference, while the ancient genomes are identified by the code of the samples from which they were assembled.

Finally, we performed a de novo reconstruction assembling multiple MAGs, among which the most abundant was identified as *Olsenella* sp. *oral taxon 807* (Supplementary Data 9, Supplementary Fig. 13). Thus we decided to further investigate this species, both for being present among multiple samples belonging to different Neolithic phases, and also for being already described as a possible microbial biomarker associated to this transition^16^. As expected, the phylogenetic analysis indicated the ancient Olsenella genomes to be closely related to the modern reference for this species, but they were split into two different clusters: one composed of four genomes reconstructed from the most ancient samples, and the other from two CA samples that were phylogenetically closer to the modern reference (Fig. 5a). Some regions were commonly missed among all of the ancient genomes (Fig. 5b), and could be related to more recent elements of evolution. Overall, a total of 123 regions were depleted in the ancient genomes, corresponding to several protein families (Supplementary Fig. 14 and Supplementary Data 10). Most parts of these regions contained only hypothetical proteins. Two large gaps, located at ~1900 kbp and ~2200 kbp, contained protein families involved in defence mechanisms and interactions with other microbial commensals: CRISPR-associated protein, antitoxin HigA, mobile element, Fic domain proteins, and integrase/recombinase, and tetracycline resistance family (TetR).

For more details regarding the genome reconstruction, please refer to the Supplementary Discussion.

## Discussion

In recent years, a more detailed picture of ancient Europe has emerged, due to joint efforts in the fields of archaeology and human genetics^34,35^. However, we are still missing some tesserae from the global mosaic. For example, knowledge regarding the evolution of the human microbiome is still elusive and fragmentary.

In the present study, we followed the evolution of human symbionts throughout the entire Neolithic period, comparing the Neolithic microbiome with that of upper-Palaeolithic hunter-gatherer and subsequent Copper Age populations. We observed different clusters that were previously undetected in the scientific literature, characterised by specific oral microbial profiles that follow the chronological evolution of these populations.

Most of the detected changes in species abundance across time could be related to shifts in subsistence strategies (such as that between hunter-gatherer and farming) or to modifications in the elements that constitute the Neolithic diet. A first distinction concerns PA samples, whose microbiomes seem to clearly differ from the subsequent Neolithic ones, from a taxonomic and functional point of view. The microbiomes of the analysed hunter-gatherer individuals are highly similar, despite belonging to a long chronological period that encompasses the last glacial maximum, and possibly a population replacement in the subsequent period^36,37^. This may be considered to be the reflection of a long-standing similar subsistence strategy based on a substantial degree of consumption of animal protein and fat, along with starchy carbohydrate foods, as suggested by combining the microremains and functional results here reported, with zooarchaeological data from Paglicci Cave^38,39^. The identified differences in the carbohydrate pathways between the PA and Neolithic samples are probably related to a variation in the selected carbohydrate sources. Indeed, microremains analysis disclosed a higher degree of taxonomic variability in the consumed starch grains among the PA samples with respect to the Neolithic samples (see Supplementary Discussion), which is in accordance with the higher abundance of the bacterial starch metabolism pathway. Interestingly, evidence of starch records attributable to oat (*Avena* cf. *barbata*) has been detected both in the dental calculus of five PA individuals (three Gravettian and two Epigravettian), and on a grinding stone unearthed in an early Gravettian layer of the same site of Paglicci Cave, providing multiple source of evidence for the consumption of this plant across all of the considered Palaeolithic phases^38^.

Conversely, the Neolithic samples were characterised by a restricted group of starch grains during all of the examined Neolithic phases, and by different taxonomic and functional variables related to carbohydrate, vitamin and amino acid metabolism as well as virulence factors.

From a taxonomic perspective, with the beginning of the Neolithic transition, species that were previously poorly attested during the PA as the pathogenic members of the red and orange complexes, along with other taxa (i.e.*, Olsenella* sp. *oral taxon 807, Parvimonas micra, Desulfomicrobium orale* and *Filifactor Alocis*, among other), began to appear or increase in abundance. Bacterial species that belong to the red and orange complexes co-aggregate together, and are known etiological agents for periodontal disease^27^. They carry several virulence factors (e.g. fimbriae and proteases) with proteolytic capabilities that are able to affect host connective tissue and trigger immune response dysfunction^40^. Recently, the presence of *P. gingivalis* has also been associated with several extra-oral inflammatory conditions such as cardiovascular disease, diabetes and Alzheimer’s, and it seems to exert a direct influence on the host gut microbial composition^41–43^. Thus, the observed increase in virulence factors across all of the Neolithic phases, and especially during the LN/CA periods, relies principally on the increased abundances of red complex species. Overall, Neolithic samples were also characterised by the presence of galactose metabolism, which could probably be related to the consumption of milk products. Galactose is metabolised from lactose, a disaccharide sugar from which milk and its derived products are primary dietary sources^44^, that are able to stimulate the growth of lactose-fermenting bacteria^45^. Actually, the identification of yeast spores from micro-debris analysis may suggest the consumption of fermented food, which could be related to a milk source. The beginning of animal secondary product exploitation is generally reported for the periods following the Neolithic^46^, but milk consumption started much earlier, as also confirmed by lipids remains on pots, as well as by archaeozoological records from contemporary Italian sites^47^. Several taxonomic and functional features were not equally distributed among the entire Neolithic period, but rather, a significant difference was found between the earlier and the later phases. Subjects that belong to the first part of the Neolithic period (i.e., EN and MN), corresponding to the Impressed Ware and Passo di Corvo cultures, share common taxonomic and functional elements and show traits that are intermediate between the two extremes constituted by PA and LN-CA. This evidence defines the early phases as a sort of “transitional state”, which could account for the lack of strong markers in previous analyses that have principally focused on the beginning of the agricultural transition^16,48^. Moreover, the proposed scenario is in accordance with stable isotope analysis, which showed that early farming communities in Central-South Italy consumed a wide range of foods, demonstrating continuity with previous hunter-gatherer communities^26^. Therefore, our result would support the hypothesis that the transition to the new subsistence strategy was gradual and regionally specific, with a strong adaptation of the arriving Neolithic people to locally available resources, instead of a drastic change to a homogenous diet, as was also reported by isotopic and botanical data, along with evidence of continuity in culinary practices in other European regions^49–51^. The site of Palagiano was the only one to demonstrate a high degree of intra-site variability, which was not associated with any anthropological metadata (e.g. age, sex or pathologies). However, it is noteworthy that archaeological evidence has pointed out the existence of different funerary models in the same site, which have been interpreted as a reflection of social differentiation^52^. Considering that most parts of the samples belonging to the same burial were grouped in the same cluster (C3) according to taxonomic analysis, we can suppose that the hypothesised social differentiations could indicate a difference in resource access or in dietary choice.

Conversely, during the second half of the Neolithic period, multiple taxonomic and functional traits led us to suppose the adoption of a high-carbohydrate/low-protein dietary regimen. The higher presence of *Capnocytophaga* spp., as well as *Ottowia HOT 894*, *Neisseria elongata* and *Rothia aerea*, which were all enriched in LN and CA samples, has been associated with a higher consumption of dietary fibre, both in the vegan salivary microbiome and in the plaque microbiome, respectively^17,19^. Moreover, the oral microbiome of the modern vegan population also demonstrates an increase in specific amino acid pathways, such as lysine, glycine and cysteine biosynthesis, which were significantly enriched even among LN and CA samples^19^. The presence of these traits suggests an increase in carbohydrate intake within the LN/CA periods, and fits with the data recorded by isotopic analyses, reporting a decreasing trend in animal protein intake and an increase in vegetal protein consumption from EN to LN and CA, along with an intensification of the agricultural practices in South-Italian farming communities at the end of the Neolithic period^25,53^. Moreover, and always during the LN period, botanical records recognised a change in carbohydrate resource selection, with a reduction in the taxonomic variability of cereals and legumes and an increase in drought-tolerant plants (e.g., barley and einkorn wheat - *Hordeum* spp. and *Triticum monococcum-*)^24^. This change in agricultural practices has been associated with specific environmental conditions, more than cultural choice, related to the occurrence of two dry climatic phases that affected South Italy between the FMN and LN periods (4500-4000 BC) and during the transition from LN to CA (4000-3500 BC)^24^ (Supplementary Fig. 15).

By combining botanical records with the cited isotopic data and our results, we thus hypothesise, that the reasons behind the alteration in microbial composition during the late Neolithic could be related to changes in dietary components (e.g., cereals and edible plants). The local populations probably applied these changes to face drier climatic conditions, which may have influenced the availability of the food plants previously consumed^24^. The dietary variations, which affected carbohydrate intake, ultimately varied the oral species abundance and the plaque microbial composition^17^.

The oral microbial community of the LN/CA samples present the highest abundance of species related to the red and orange complexes and of virulence factors, suggesting that the oral ecosystems of the LN and CA populations suffered a worse state of health than those of their recent ancestors. In this frame, it is noteworthy that anthropological analyses, mainly retrieved from published articles (see Supplementary. Discussion), reported that most of the considered samples for LN and CA periods were characterised by a higher incidence of oral diseases. Conversely, the PA samples demonstrated good oral health conditions, while the EN/MN samples accounted for a low incidence of periodontitis and caries (Supplementary Data 1). This trend is also in agreement with previous studies on skeletal remains, which already suggested a decline in oral health condition associated with the transition to agriculture^54,55^.

Considering additional aspects of Neolithic populations’ health conditions, we detected the presence of diatoms, suggesting the consumption of polluted water, which could have had important implications for human health^56^.

Finally, additional factors explaining the observed changes in bacterial ecology may be related to genetic variation across symbiont genome evolution, which could have affected species interaction and adaptation. Bacteria living within the same community share principally negative interactions (i.e. competition) instead of positive ones (i.e. cooperation), especially if they live in a highly biodiverse environment where the competition for nutrients becomes stronger^57^. Antibacterial toxins, along with other virulence factors, are the weapons used by species to compete, creating strong negative interactions among them^58^. The analysis of the ancient *Olsenella* sp. *oral taxon 807* genomes detected a number of missing elements, mostly related to virulence factors and biofilm formation, compared to the modern ones, suggesting that its ability to compete and interact with other oral species living in the same niche has evolved recently. This, together with the stronger selective pressure exerted by dietary changes, would ultimately have affected the oral microbiome ecology.

In conclusion, our analysis provides evidence for the presence of two main shifts affecting the abundance of several species of oral microbiome in the investigated populations: (i) the first is related to the cultural transition associated with the introduction of the agricultural lifestyle in this region, which drove an initial modification in the microbiome composition while retaining some aspects of the previous hunter-gatherer samples; (ii) the second, occurred during the late Neolithic, and is probably linked to the adaptation to distinct dietary elements, when the changes observed in the first phase became more prominent and some species that were strongly present in the hunter-gatherers almost disappeared, probably due to the progressive adaptation of farmers to distinct dietary elements.

## Methods

### Sample collection and sequencing

In agreement with the local Superintendence of Cultural Heritage, 79 samples were collected from multiple archaeological sites in the same geographic area (Central Southern Italy) where the Neolithic period started ~6200 BC, in the peninsula. In order to obtain an in-depth understanding of the entire Neolithic period, we collected samples that covered the entire transition, from its beginning to its end, following the main archaeological and cultural subdivisions of this period: early Neolithic (EN, corresponding roughly to the Impressed Ware culture, from the VII to VI millennia BC), middle Neolithic (MN, corresponding to the Passo di Corvo painted ware, during the VI millennia BC), final part of the middle Neolithic (FMN, corresponding to the Serra d’Alto painted ware from the end of the VI to halfway through the V millennia BC), late Neolithic (LN, corresponding to Diana ware, from the second half of the V to the beginning of the IV millennia BC). Moreover, we considered both the previous hunter-gatherer individuals from the same area at the site of Paglicci Cave (PA, corresponding to the upper-Palaeolithic Gravettian and Epigravettian cultural contexts) and the post-Neolithic phase, called the Copper Age (CA, corresponding to Gaudo and Laterza ware). In order to contrast any potential biases related to the oral biogeography, the molar dental calculi, when available, were mainly sampled (Supplementary Table 1)^59^. More information regarding the investigated archaeological site, the cultural background and radiocarbon dating are reported in Supplementary Table 1 and Supplementary Methods.

DNA extraction and sequencing were conducted on 76 samples in a dedicated aDNA clean laboratory at the Department of Biology of the University of Florence (Italy), following validated guidelines to avoid modern DNA contamination^60^. After decontamination under UV light for 10 minutes, calculus was coarsely ground with a sterile micropestle and treated accordingly to Procedure A in Modi et al.^61^ which permits the joint analysis of genetic material and food residues from the same calculus sample. Briefly, after overnight digestion in 1 mL of extraction buffer (0.45 M EDTA pH 8.0, 0.25 mg/mL Proteinase K, 0.05% Tween 20), residual pellets were stored for microremains analysis, while DNA was extracted using a specific silica spin-column protocol designed to recover short molecules^62^. After centrifuge at maximum speed for 2 min, supernatant was incubated with 10 mL of Binding Buffer (5 M Guanidine hydrochloride, 0.05% Tween 20, 40% 2-Propanol) and 400 µL of 3 M Sodium Acetate pH 5.2 into silica columns (High Pure Viral Nucleic Acid Large Volume Kit, Roche Diagnostic – Mannheim, Germany). After two washing steps with Wash Buffer (High Pure Viral Nucleic Acid Large Volume Kit, Roche Diagnostic – Mannheim, Germany), DNA was eluted twice in 50 µL of TET buffer (1 mM EDTA pH 8.0, 10 mM Tris-HCl pH 8.0, 0.05% Tween 20). Twenty microliters of each extract were then converted in double-indexed Illumina libraries^63,64^ with no uracil DNA glycosylase (UDG) treatment in order to retain nucleotide misincorporations. A unique combination of two 8-base long indexes was added to both ends of each library molecules by 10 cycles of PCR. The entire library volume was split in 4 reactions and was used as templates in 100 µl PCR reactions containing 1x Pfu reaction Buffer, 2.5 U Pfu Turbo DNA polymerase (Agilent Technologies – Santa Clara CA, USA), 250 µM each dNTP and 200 nM each indexing primers (P5 primer: AATGATACGGCGACCACCGAGATCTACACxxxxxxxxACACTCTTTCCCTACACGACGCTCTT; P7 primer: CAAGCAGAAGACGGCATACGAGATxxxxxxxxGTGACTGGAGTTCAGACGTGT). Cycling conditions were comprised of an activation step lasting 2 min at 95 °C, followed by 10 cycles of denaturation at 95 °C for 30 s, annealing at 58 °C for 30 s and elongation at 72 °C for 1 min, with a final extension step at 72 °C for 10 min. PCR products were purified using the MinElute PCR Purification Kit (QIAGEN – Hilden, Germany) and eluted in 25 µl TET buffer. After a qualitative and quantitative check with Agilent TapeStation (D1000 ScreenTape and reagents, Agilent Technologies – Santa Clara, CA, USA), libraries were pooled in equimolar amounts and sequenced on an Illumina NovaSeq 6000 platform in paired-end mode with 2 × 51 + 8 + 8 cycles^65^.

### Microscopic analysis

Microscopic analysis was conducted on 27 samples by the Laboratory of Palinology at the University of Florence for the Palaeolithic samples, and at the DANTE-Diet and ANcient TEchnology laboratory of the Sapienza University of Rome, for the Neolithic and Copper Age samples. Based on our experience, it is possible to retrieve and analyse the microremains, starting from the sample pellet used to extract aDNA in a proper clean lab^61^. Moreover, six samples were analysed for both sources (i.e., dental calculus and pellet), to further compare the results obtained.

Dental calculus sampling was conducted following the protocol systematised by Sabin and Fellow Yates^66^, with some variation (e.g., disposable blades were changed after each sample extraction). Extraction and decontamination procedures followed standard published protocols^66–68^, and were conducted in dedicated clean spaces not connected to modern botanical work, and under strict environmental monitoring. Cleaning was conducted daily in order to prevent any type of modern contamination. Bench space surfaces were cleaned prior to the analysis of each sample, using soap and ethanol, covered by aluminium foil and using clean, starch-free nitrile gloves at all times. Calculus cleaning was conducted under a stereomicroscope, on a previously washed Petri dish, with magnifications of up to 100×. The removal of mineralised soil adhering to the surface of the calculus was meticulously performed, using sterile tweezers to hold the sample, and a fine acupuncture needle to gently scrape off the soil attached to the external layer of the mineralised plaque. The procedure was performed using drops of 0.5 M HCl acid to dissolve the mineralised flecks of soil, and ultrapure water to block the demineralization, as well as to wash and to remove the contaminants. The mineralised soil removed was stored in plastic tubes for monitoring analysis. Then, the clean calculus was washed in ultrapure water up to three times, to remove any trace of loose sediment, dissolved in a solution of 0.5 M HCl, and subsequently mounted on slides using a solution of 50:50 glycerol and ultrapure water. Furthermore, control samples from the clean working tables and dust-traps were collected and analysed for comparative purposes, in order to prevent any type of modern contamination. This is a practice that is routinely performed in our laboratories, even at times when no archaeological analysis occurs, to allow for a better understanding of the flow of contaminations through the seasons. We did not retrieve any debris that was morphologically similar to any of the remains in the environmental control samples. Starch grains amounted to a very minor fraction of the laboratory ‘dust’. The pellets were treated with 10% HCl for 12 h and included in a 50% *v*/*v* water–glycerine solution. The analysis was performed under light microscopy, under bright-field light and cross-polarised light. Micro-debris embedded in the calculus matrix and in the pellet were analysed using a Zeiss Imager2, with magnifications ranging from 100× to 630×.

For the identification of archaeological starch grains, a modern reference collection of 300 plants native to the Mediterranean region and Europe was used, along with the published literature, and with the *Herbarium Centrale Italicum* (FI) and samples collected from the Botanical Garden of the University of Florence^30,38^. Spores were identified by comparison with the available literature^69^. The groups of grains were counted as a presence. Phytolith nomenclature was according to ICBN 2.0.

### Metagenomic sequence processing and Taxonomic analysis

Metagenomic sequences were processed for their quality, and the adaptor sequences were trimmed and paired-end reads were collapsed using AdapterRemoval (v2.2.0)^70^ software with the following options: *-*minlength 30 –minquality 25 –trimns –trimqualities –collapse. Then, sequences duplicates were removed using Prinseq^71^ (v 0.19.1) (https://prinseq.sourceforge.net/) and analysed with Kraken2 (v.2.0.8-beta) for taxonomic identification^72^. We created a custom database, updated to December 2020, of bacterial, viral, archaeal and mitochondrial genomes from the NCBI Reference Sequence (RefSeq) database (https://www.ncbi.nlm.gov/refseq/). To avoid spurious classification, we masked the reference genomes for low-complexity regions with Dustmasker (v.1.0.0) (https://www.ncbi.nlm.nih.gov/books/NBK569845/). The output of Kraken2 was then analysed through Bracken software (v.2.5) to estimate the species read abundances^73^ (Supplementary Data 11). This same approach has been used for the reference modern and ancient microbiome metagenomic samples used for exploratory comparative analysis and reported in Supplementary Table 3. We selected the species that were present at a relative abundance of higher than 0.02 in the whole dataset. This type of taxonomic filter has been advocate to reduce the possible influence of low-abundance species and singletons present in the dataset, and it is more suitable than the classical rarefaction approach^74,75^. Thanks to the applied threshold, we obtained 49 species that were subsequently validated for their deamination profile and thus confirm their antiquity. We aligned each sample reads to its respective reference genomes deposited in the NCBI RefSeq, database using only the species reported as the “reference” or “representative”. The Burrows-Wheeler Alignment (BWA v.0.7.15)^76^ programme was used for this aim, using the *aln* algorithm with high stringency (*-n 0.1*). Aligned sequences were then investigated for their cytosine deamination profile using PMDtools (v.0.60) (–threshold 1 –requiremapq=30) (https://github.com/pontussk/PMDtools). For each of the species aligned, we converted the bam files to bed using bedtools (https://bedtools.readthedocs.io/en/latest/), and estimated the edit distance (-tag NM) that was subsequently used to compute the edit distance algorithm (–Δ%), to further confirm sequence authenticity^77^. As reported before, we evaluated –Δ% > 0.8 as being authentic, to assess for possible horizontal transfer events or the presence of genetically closely related species within the samples’ microbiome^78^. All of the information regarding the deamination profile and –Δ% for each species and all samples are reported on Supplementary Table 4. We further evaluated the reads fragmentation level and differences in the deamination profile across all of the different periods here investigated, to observe the effect of taphonomic events over time (Supplementary Figs. 4 and 5).

To investigate the extent of possible modern contamination, and to authenticate the oral sources of our ancient samples, we compared the obtained microbiome profiles with those of both modern and ancient metagenomic samples. Modern faecal, soil, skin and oral (both dental calculus and plaque) microbiomes were retrieved from European Nucleotide Archive, while new environmental and laboratory controls were generated (Supplementary Table 3). Two soil samples directly sampled in the Apulia and Marche regions, and with two laboratory controls (i.e., from the extraction and sequencing processes). Moreover, previously reported ancient oral microbiomes from other studies were added as further controls. We investigated the positions of our samples with respect to modern and ancient references through a nonmetric multidimensional scaling of Bray-Curtis distances, based on species distribution using phyloseq (v.1.30.0) package from R software (v. 3.6.3) (Supplementary Discussion). Moreover, we estimated the percentage of the oral source in our dataset, using Sourcetracker (https://github.com/danknights/sourcetracker), considering a subset of modern microbiomes obtained from shotgun metagenomic using the Illumina platform, and having >10 million reads as sources (SI, Supplementary Fig. 2). Samples demonstrating high oral source (>75%) were retained for the analysis. Finally, in order to eliminate possible contaminants, we compare the identified species with the Human Oral Microbiome Database (HOMD) to assure the selection of known human oral species. In this sense, the only questioned species was *Burkholderia pseudomallei* which is an environmental bacterium and the causative agent of melioidosis in humans^79^. The genus *Burkholderia* have been detected in human oral cavity and in the dental plaque^80,81^, but no evidence of *B. pseudomallei* have been described previously for dental calculus thus we cannot exclude that its presence could be related to an ancient contaminant or not.

The Bracken output was used to investigate species distribution among samples using R software (v. 3.6.3). First, data were normalised through a centred log-ratio (clr) transformation, introduced by Aitchison, using the microViz package (v.0.9.0). This transformation makes the data symmetric, scale-invariant and linearly related, putting the data in a log-ratio coordinate space^82^. This approach is one of the most well-suited for normalizing compositional data^83^. To explore the existence of any possible groups within our cohort through an unsupervised approach, we performed the gap statistic at the genus level, which is a standard method that determines the number of clusters in a dataset^84^. We applied the clusGap function from the cluster package (v.2.1.0)^85^ to calculate the goodness of the clustering measure on a phyloseq object (v.1.30.0)^86^ with a bootstrap of 100. Then, we used NetCoMi (v.1.0.2)^87^ to generate a network to investigate the relationship shared by samples based on the Aitchison distance at genus level which is more stable for subsetting and aggregation of the data, and for being a true linear distance^83^. Data were sparsified with the k-nearest neighbour (*k-nn*) algorithm, and a hierarchical clustering analysis was applied, using the average linkage method and the *k* value obtained by the gap statistic (method = “average”, k = 5). We used network analysis to highlight the connections among the samples (not just their distance), and identified existing clusters based on samples’ microbiome composition. To further corroborate this approach with a more classical analysis, we performed PCoA using Aitchison distance, and performed PERMANOVA and ANOSIM to validate cluster subdivision identified by network; we also checked for the possible influence of read counts, extraction and library batch. We applied pairwise Adonis (https://github.com/pmartinezarbizu/pairwiseAdonis) to highlight the differences between pairs of clusters.

In order to understand which of the few available metadata (i.e., the period, site, age of death or sex) may have played a major role in cluster distinction, we applied a random-forest classification with 100 trees, using the radiant package (v.1.4.0) (https://github.com/radiant-rstats/docs). Random forest identified the relative influence of each feature on the created model to classify the cluster outcome. To validate the classification model obtained from random forest analysis, we performed an association test using the *assoc* function from the vcd package (v. 1.4-9) which computes Pearson’s chi-squared (X^2^) value, which is designed to evaluate the deviation from an independence model in categorical data.

To explore and to identify the species that significantly changed in abundance across time, we applied a double approach. We compared clusters composition using DESeq2 (v.1.26.0), accepting as significant only those species that showed an adjusted *p* < 0.05 and that showed significance from more than one comparison, to avoid possible spurious significant results. Then, to highlight the strongest associations of species with clusters and period variables, we performed a multivariate analysis by linear model (MaAsLin v.1.0). This statistical framework applies a general linear model to detect the associations between clinical metadata and microbial taxa, and it is widely used in modern epidemiological studies^88^. Parameters that were applied for this analysis were as follows: a maximum false discovery rate (significance threshold) equal to 0.05; a minimum for feature relative abundance filtering equal to 0.001; a minimum for feature prevalence filtering equal to 0.01. Differentially abundant species were regrouped based on their membership with the ecological complexes that constituted the oral microbial community^27^.

In addition to the taxonomic profile, we evaluated whether changes in microbiome composition may correspond to differences in functional profile through an analysis of gene content performed with HUMAnN 2.0^89^. We assessed the gene content profile in our samples, and regrouped them using UniRef90_ko. In order to avoid bias in gene content related to modern contaminants, the obtained profiles were filtered, selecting the species that were previously validated to be authentically ancient. We compared the obtained KEGG (Kyoto Encyclopedia of Genes and Genomes) orthologs distributions using LEfSe (v.1.0)^90^ and selected only results with *p* < 0.05 and LDA higher than 2. Regarding the identification of the virulence factor (VF), genes detected as being significant were matched against a custom database containing information from the Virulence Factor Database (VFDB) generated by Chen et al.^91^.

Overall, plots were produced using ggplot2 (v.3.3.5)^92^, with colour blind friendly palette^93^.

### De novo genome assembly

Reconstruction of an ancient oral genome proceeded following the Wibowo et al. pipeline^9^ for contigs validation. Each sample was assembled using SPAdes^94^ (v.3.15.3) with the *--meta* option. Each sample was mapped against the relative assembled contigs using Bowtie2 (v.2.3.5.1)^95^ with the default settings. The outputs were indexed and sorted through SAMtools (v.1.9)^96^ and the generated BAM files were sequentially binned using MetaBAT 2 (v.2.12.1)^97^. An evaluation of assembly quality was performed via a lineage-specific workflow in CheckM (v.1.1.6)^98^, testing for genome completeness, contamination level, coverage, N50 values, mean contig length and size of the largest contig (*see* Supplementary Table S5). The assembled contigs were then used for taxonomic identification with Kraken2. Only genomes with a level of completeness of higher than 85%, a contamination level <5% and a taxonomic identification >95% were considered for the following analysis. Finally, to assess for the aDNA damage profile the *damageprofiler.sh* script from Wibowo et al. was used but the last part of the script was modified using PMDtools. A plot of the deamination profile for each sample was reported in Supplementary Fig. 13. Contigs alignment to the reference was performed with BLAST Ring Image Generator (BRIG) (v.0.95)^99^, using upper and lower identity thresholds of 70% and 30%, respectively. This software was used to generate both Fig. 5 and Supplementary Fig. 14, this latter contain the reference genetic annotation retrieved by NCBI database.

Contigs were then uploaded to PATRIC (v.3.6.12)^100^ for further annotation, phylogenetic and proteome analysis. Particularly, all of the six reconstructed genomes were annotated for their genomic features within the PATRIC workspace using RASTtk^101^. The phylogenetic tree was constructed through RAxML (v.8.2.11)^102^, using 23 modern genomes references and 159 genes. A proteome Comparison tool was then employed to investigate ancient protein similarity with the mother reference, and to identify functions and missing regions. It uses the BLASTP algorithm to compare protein sequences as either being unique, a unidirectional best hit or a bidirectional best hit when compared to the reference genome. The Protein Family Sorter tool was used to investigate the distribution of protein families that cross the genus boundary (PGFs) among ancient samples with respect to the modern reference.

### Reporting summary

Further information on research design is available in the Nature Portfolio Reporting Summary linked to this article.

## Supplementary information

Supplementary Information

Description of Additional Supplementary Files

Supplementary Data 1

Supplementary Data 2

Supplementary Data 3

Supplementary Data 4

Supplementary Data 5

Supplementary Data 6

Supplementary Data 7

Supplementary Data 8

Supplementary Data 9

Supplementary Data 10

Supplementary Data 11

Reporting Summary

## Data Availability

All the sequence data generated for this study have been deposited in the NCBI Sequence Read Archive (SRA) under BioProject PRJNA791766. Processed data, microscopy data as well as genome assembly and annotation data are contained within the paper and its Supplementary Data files. Archaeological samples were collected at the “Soprintendenza Archeologia, Belle Arti e Paesaggio per la Città metropolitana di Bari” in Bari (Italy), at the “Soprintendenza Archeologia, Belle Arti e Paesaggio per le Province di Barletta- Andria -Trani e Foggia” in Foggia (Italy), at the “Soprintendenza Archeologia, Belle Arti e Paesaggio delle Marche” in Ancona (Italy), at the “Museo delle origini” at the University “La Sapienza” in Rome (Italy), and at the museum “Museo delle Civiltà” in Rome (Italy). Source data are provided with this paper.

## Source data

Source Data

## Code availability

Code is available at https://github.com/AndreaQ7/HuME and at Zenodo^103^ (10.5281/zenodo.7198970).
