# Supplementary material for: Ancient oral microbiomes support gradual Neolithic dietary shifts towards agriculture
Source: Nat Commun. 2022 Nov 22;13:6927. doi: 10.1038/s41467-022-34416-0 (PMC9681849; doi:10.1038/s41467-022-34416-0)
Supplement: Supplementary file 1 — Supplementary Information [file 41467_2022_34416_MOESM1_ESM.pdf]

Ancient oral microbiomes support gradual Neolithic dietary shifts towards agriculture

Quagliariello *et al.*

Supplementary Information Text

Supplementary Methods

Description of archaeological sites

Paglicci (Apulia)

The Palaeolithic cave-site of Paglicci is located on the Gargano Promontory (Apulia, Foggia, about 143 m above sea level). After the first fieldworks directed by the Natural History Museum of Verona (F. Zorzi dir.), from 1971 onwards, research was resumed by the University of Siena (A. Palma di Cesnola and A. Ronchitelli dirs.). The Upper Palaeolithic stratigraphic sequence is 12 m thick and embraces the whole chrono-cultural succession known for the area, spanning from the Protoaurignacian to the Final Epigravettian[1]. Several mobiliary art objects were found in the Gravettian and (most of them) in the Epigravettian layers. In addition, Paglicci yielded the only Palaeolithic wall paintings discovered in Italy so far. These are located in an inner room and consist of horses and hands, probably attributable to the Ancient Epigravettian[2].

The human remains analysed in this paper are from different stratigraphic units:

PA 133 is an isolated lower third molar from layer 23C2 (beginning of the Early Gravettian)[3], characterised by the presence of a tephra dated to 33000 years ago[4]. PA 12 is the burial of a young individual (aged 12-13 years) found on the top of layer 22A[5]. PA 25 is the burial of an 18–20-year-old woman related to the Evolved Gravettian[6,7]. PA 14 B is a mandibular fragment from layer 20b (Evolved Gravettian). All these specimens were found in the main excavation trench. The other specimens (maxillary fragments PA 140 and 157, mandibular fragments PA 38 and PA39, and the lower premolar PA 150) are from a part of the deposit located between the cave's atrium and the second chamber and belonging to the Epigravettian[3]. Except for the general chronological indications provided for the Epigravettian remains, dates reported in the Supplementary Data 1 are calibrated with the new IntCal20 curve in the OxCal 4.4 software[8,9]. Overall the general oral condition of Paglicci samples was good, with almost no presence of dental pathologies detected, thus confirming a general good health condition of the entire group[10].

Biccari (Apulia)

The site of Biccari is located in the area of the Dauni mountains, in the north of Apulia region. Numerous preventive archaeological interventions linked to the exploitation of wind energy in the area, have brought to light many traces of attendance of the area during the more ancient phases of Neolithic. The site of Biccari is characterize by the presence of various dwelling (*i.e.,* huts) with elliptical shape and with vegetable framed walls. The interior space was occupied by a fireplace. Inside the site were also found two-fired furnaces, used for cooking food and ceramic[11]. One of the two tombs detected during the excavations was considered for the present study basing on the good conservation level. The body of a mature woman, between 30 and 40 y.o., was placed inside a hypogeum called "*Ipogeo della signora*" (literally: Hypogeum of the lady) which had an oval plan and presented various ritual elements in the vicinity of the body. The skeleton was stretched out on the left flank, the hands close to each other and adjacent to the head, with the face turned to the east. In proximity to the feet, elbows and head stone blocks were arranged to support body position. A point of flint was placed on the right heel, a neck vase was instead placed near the head. During the funeral ritual they had scattered ceramic fragments around the woman's body along with ash, coals and even reddish ochre. The body, dated between 5740-5620 BC, shows that the woman was engaged in repeated and exhausting activities, suggesting that men and women were both engaged in the work activities of the community. The teeth did not show any caries or oral specific disease although very worn on the occlusal plane and also showed signs of extra-masticatory wear[12].

Portonovo (Marche)

The site of Portonovo-Fosso Fontanaccia, is located along the Adriatic coast of Marche region of the Italian peninsula. This site is characterized by the unique presence of 23 ovens made at different times and probably never used at the same time. Radiocarbon dates and archaeomagnetism indicate a long-term occupation of the site from 5900 to 5200 cal. BC[1],[2] corresponding to Early Neolithic of the mid-Adriatic regions of Italy. The discovery of paleobotanical remains near and inside the ovens (principally *Hordeum vulgare* and *Triticum dicoccum*), supports the hypothesis of their use for the roasting of cereals as a pre-treatment for their conservation and consumption[13]. Anthropological analysis on the skeletal remains here analysed, found a low frequency of caries and other dento-alveolar disease as well for enamel hypoplasia, thus suggesting a good oral health status[14]. Moreover, even isotopic analysis has demonstrated an evident dependence on animal products (especially herbivore mammals) and even a moderate consumption of marine fish[15]. Samples belonging to this site were analysed through isotopes analysis in Lelli et al.[16]

Passo di Corvo (Apulia)

The site of Passo di Corvo is placed on a terrace of the Amendola plain, close to the cities of Foggia and Manfredonia in the Apulia region. The archaeological site was principally investigated between the 1960s and '80s[17]. The extension of the village is around 40 ha, with several C-shaped ditches reported with widths at the

mouth from 4.6 to 6.1 m[17]. Two areas of the site were used for funerary aim, reporting at least 12 individuals, with one more female subject found directly inside one of the mentioned ditches. The functions of the numerous C-shaped ditches are still debated but, among other interpretation, it was supposed to have favoured groups' identity with some ritual or social cohesion aim[18]. Overall, the site was interpreted as a residential context datable to the early phases of the Middle Neolithic and it has been estimated to be occupied by a large number of sedentary families (around 30-35), thriving on agricultural economy but with herding contribution constitutes by cattle, ovicaprids and pigs[19]. The strontium isotopic analysis indicates that all the analyzed individuals have a local origin [20]. A typical ceramic style diffused in the Middle Neolithic across Southern Italy, characterized by painted pottery, takes its name from this site. Two main styles are recognized: one called "archaic Passo di Corvo", which is characterized by painted white bands; and a more recent style called "typical Passo di Corvo", that stands out for clay pots painted with simple straight or curved red bands and geometric motifs. The site has been radiocarbon dated to 5570-5470 BC[21]. The burials are datable to the middle Neolithic and are interpreted as single primary inhumations. Most bodies were lying in a contracted position on one side. Interestingly, a number of *peri mortem* damage and cut marks have been identified on several bones in this site, shading light on *post-mortem* operations on the intact or partly decomposed body[22]. This practice has been observed even on other site in the same area, such as Masseria Candelaro, and may be linked to ritual and symbolic aspects[23].

Masseria Candelaro (Apulia)
Masseria Candelaro is one of the many entrenched villages of the Tavoliere area in Apulia region. It is located at the basis of Mount Aquilone (between 25 and 30 m above sea level) and close to the river Candelaro. After a first excavation campaign during the 1978, it was more intensively investigated between second half of 80's and the beginning of 90's. The site is characterized by a wide sequence of occupation since the ancient Neolithic until the end of middle Neolithic, during which considerable structure were built such as: ditches, trenches, landfill area and burials. The best preserved structures are the fence moats, with the smaller one (moat Z) that delimits a settlement attributable to the ancient Neolithic (called Candelaro phase I): at its basis, indeed, were found several ceramics element belonging to Guadone style. After this ancient period, when the moat was largely obliterated, archaeologists found evidence of a new phase called Candelaro II with ceramics referable to Passo di Corvo style. Samples collected for this study belong to this second phase. This same chronological phase corresponds to an expansion of the village with the excavation of three new concentric ditches dated to 5400-4850 BC[17,23,24]. A large archaeozoological analysis of this last phase, testify the presence of several young animals (less than 4 y.o.). This exploitation model, motivated by the exclusive supply of meat, appears fairly standardized within the different Neolithic contexts analysed of this period. Most part of animal were linked to farming activity but with a good integration of hunted animal such as hare (*Lepus Europeus*), roe deer (*Capreolus capreolus*), marten (*Martes cfr. martes*), turtle (*Testudo hermanni*) and birds' remain (*Aves ind*.)[23]. Samples belonging to this site were analysed through Carbon and Nitrogen isotopic ratio by Lelli et al. for dietary reconstruction [16] and through Strontium isotopes for individual mobility by Tafuri et al.[20]. Anthropological analysis highlighted the presence of 23 individuals with a good oral health status: only 6 samples presented one caries; slight dental calculus presence; slight hypoplasia insurgent around 3-4 years old[23].

Palagiano (Apulia)
The site of Palagiano overlooks the plain of Taranto. To date, about 380 square meters have been investigated that have brought to light a small funerary area. Ten graves "a grotticella" have been identified, chronologically dated to the half of V millennia BC[25]. From the architecture of the grave structures, rather than from the ceramic items, it is clear the degree of importance of the investigated burials and the considerable investment of resources by the community. The access to the funeral area was indicated by a pair of stone markers. The bodies were laid on stone slabs and in a semi-contracted position with N-S orientation. Traces of cinnabar have been found on some bodies and also on part of the trousseau, probably remains of funeral rituals. Several ceramic items belonging to Serra d'Alto culture have been found in tombs 5 and 7, associated with female subjects, along with Diana "olla" bowls, and bone items. Within the "olla" bowls in tomb 5, traces of animal fat and conifer resin (*Pinus* sp.) were present[26]. Within the site, it is possible to find different funerary models based on body position or for the presence of specific funerary elements. These difference in funerary practices have been interpreted by the archaeologists as a reflection of possible social differences within the same community[25].

Ripatetta (Apulia)
Recent archaeological investigations (between 2019 and 2020) in the site of Ripatetta, in the area of Lucera (Foggia), were carried out for the expansion of the quarry for the extraction of clay. The excavations have unearthed numerous archaeological evidence belonging to three distinct phases: phase 1, related to the late Neolithic period (end of the V millennia B.C.); phase 2 attributable to Daunis' population between VII/VI and IV century B.C.; phase 3 of middle age period.

To the late Neolithic phase belongs a large graveyard containing 33 burials, placed on the clay soil of this area. There are at least two kind of burials within the whole area: the simple graves and the so called “grotticella” tombs. The entrance of the tombs is often buffered with large limestone slabs. Most part of the burials are single but even multiple are recorded even if at small number. Almost all the individuals recovered are adult and both female and male are present. Several grave goods were identified as belonging to the Diana culture basing on the ceramic remains, placed in the burials together with flint and obsidian remains which are more often present in male graves. Interestingly, 14 tombs were characterized by the presence of red pigment directly on the skeletal remains and in the surrounding area. Other unusual features include the metacarpal of a cervid located in a female tomb and some necklace made of black stones around the cervical vertebrae of some female subjects[27].

Deliceto (Apulia)
In 2018, during excavations for a power line, four middle Copper Age burials were discovered. They are four “grotticella” tombs, excavated in the calcarenitic layer. The burial chamber has a triangular plan, with the back wall curved and the side rectilinear. The bodies were laid on a very compact layer of beaten ground. The skeletal remains were in a very bad conservation status, indeed two of the samples were deeply contaminated by soil microbes as highlighted by SourceTracker analysis (see Supplementary Data 1)

Melfi (Basilicata)
In 2010, during excavations for a power line in the area San Nicola degli Abruzzi, two Copper Age burials were discovered. The area is part of the city of Melfi, in the Basilicata region. The two burials have been identified as belonging to the Copper Age period based on some items found within the tombs, in proximity of the skeletal remains but the anthropological and archaeological investigations are in progress.

Fermo (Marche)
Excavation campaign between the 2018 and 2019 for the hospital construction, have brought to light nine burials with variable conservation conditions and a shape close to the so called “grotticella” tomb typical of this period in this part of the peninsula. The bodies were laid curled with orientation N-S and were probably placed in a shroud or with a perishable material structure. Within the burials several animal bones (*i.e.*, sheep and pigs) were deposed probably as funeral offering, together with different ceramics and flint weapons[28].

Recanati-Cava Kock (Marche)
Two human remains were founded in 1940 at Fontanoce-Cava Kock (Recanati), in the Adriatic coast of the Marche region, and are preserved in the “Museo delle Origini” at the University of Rome “La Sapienza”. We collected dental calculus just for one of the two samples (t.B) which have been dated to 3095±80 BC at C^{14}, thus referring to an ancient phase of Copper Age period. The individual was buried in a pit dug in a clay formation and was laying on a side. The sample, identified as a 30-40 y.o. male from anthropological analysis, was buried together with an ovoid olla behind the head. The study of the skeletal remains highlighted the presence two tooth loss and a particular destructive decay, as well as enamel hypoplasia, indicating nutritional stress during childhood[29].

Troia (Apulia)
In the area of Torre de Rubeis (in the city of Troia) near the Sannoro torrent, one Copper Age burial with “grotticella” style was found. Within the tomb were located four individuals: two children, one adult and one young person under the age of 30, in a curled position. In proximity of each skull, was laid a vase and a decorated “olla”, a bowl, a glass of the Gaudo and Laterza facies. Some aspects of the archaeological remains are found even in the ceramic elements found within the sites of Valle Sbernia (Peschici – FG) and Grotta Nisco at Cassano Murge (BA)[30].

Bandita San Pantaleo - Tarquinia (Latium)
In the middle of 60’s, during geographical surveys, was accidentally discovered a Copper Age rock-cut tomb (so called “oven” tomb), dug into the tuff bench in the territory of Bandita S. Pantaleo, north of Tarquinia. In the same area were found other archaeological remains such as lithic weapons “grotticella” tombs and ceramics, suggesting the hypothesis of a significant occupation of this area during Copper Age. The burial was composed by a group of five individuals accumulated together and shuffled. The funeral equipment was composed of some flint arrow-heads, a "flask" vessel possibly used for the transport of liquids and various ceramic fragments referable to clay vessels belonging to the so called Rinaldone culture common in the area of Latium and Tuscany[31].

Gricignano (Campania)
The pluristratified site of Gricignano d’Aversa was discovered in 1998 during the construction of a US Navy logistic site area. The archaeological excavations led to the discovery of several housing structures and burial

plots dated from the final phase of the Copper Age period, corresponding to the Laterza culture, until the early Bronze Age (i.e., 2500-1800 BCE)[32,33]. Anthropological analysis on more than 200 individuals revealed a high frequency of dental calculus among all the population (75.6%), equally distributed between sexes and with small occurrence even at very young ages (5-10 y.o.)[34]. Furthermore, dental analysis indicates the use of teeth for extra-masticatory functions, with a high prevalence in females (62.2%) and just one case among males, potentially indicating distinct gender roles. Both location and morphology of the modifications point to yarn production, also corroborated by the identification of hemp fibers included in the dental calculus of two individuals[34]. Overall, samples from this site were characterized by an high incidence of dental calculus, with a mean around 75% of the population, without difference between sexes and equally distributed among all the age classes, even at young ages[34].

Supplementary Discussion

Ancient DNA validation

After taxonomic identification through Kraken2 and Bracken (see the Materials and Methods section), all the major taxonomic species (relative abundance > 0.02) detected for each sample were aligned to their respective references deposited in the NCBI RefSeq database using only species reported as “reference” or “representative”. The percentages of deaminated bases at the 5' and 3' are represented on Supplementary Figure 3 and have already been reported in Supplementary Data 4, together with –Δ%. In order to evaluate the degree of DNA preservation in all of the considered archaeological sites and periods, we calculated wheatear the differences in the deamination profile at the 5' were present among the different periods considered in this study, using the Wilcoxon test (Supplementary Figure 4).

No differences in deamination rates were detected by the Wilcoxon test, thus indicating a very similar profile of deamination, not only between the Neolithic and Copper Age samples, but even with the Palaeolithic subjects. This indicates an overall good degree of DNA preservation in the Paglicci cave samples, which may be due to the particular taphonomic condition present in the cave, as observed even for other samples belonging to similar environmental conditions[35]. We even screened fragment length, which is another maker that is associated with aDNA degradation level, and a highly fragmented profile was obtained, as expected. It is interesting to observe that samples that belong to the same period, even if from different sites, showed a similar fragmentation trend. Palaeolithic samples are characterised by a slightly more fragmented profile, as is observable for the number of reads between 30 to 40 bp (Supplementary Figure 5).

Microbiome validation and analysis

Thanks to the applied threshold described in the Methods section, we obtained 49 species that were subsequently validated for their deamination profile and thus confirm their antiquity. The Bray-Curtis distance was computed among all of the constituted dataset and analysed through a principal coordinates analysis (PCoA) to highlight the dissimilarity level with respect to other microbiome sources (Supplementary Figure 5). The results clearly show that the ancient samples presented here clustered together with other published ancient dental calculus, and bear close relation to modern oral microbiomes on the first axis. However, five ancient samples are close to modern soil and skin microbiomes; thus, we further investigated this through SourceTracker to check more precisely the contamination levels for each sample (Supplementary Figure 6).

Overall, the samples showed a very high level of oral microbiome source (>90%), while, as suspected from the PCoA analysis, only a few samples reported soil contamination (see Supplementary Data 1 for details regarding the percentages). Thus, these samples were removed from subsequent analyses.

To inspect the relations among the samples collected in this study, and to identify possible clusters, we performed a network analysis using Aitchison distance. This is a Euclidean distance between the samples after clr transformation. The Aitchison distance is more suitable for compositional data, being more stable to subsetting and for being a true linear distance with respect to the classical Bray-Curtis[36,37]. We applied hierarchical clustering on the relation between the samples identified by the network itself, obtaining 5 different clusters. In order to obtain a statistical significance that is associated with the identified clusters, we repeated our analysis, always using the Aitchison distance on a PCoA, allowing us to perform PERMANOVA and ANOSIM, using Cluster, Period, Site, Extraction Batch, Library Batch and the number of reads as variables to test (Supplementary Figure 6). We obtained a significant *p*-value associated with the Clusters and Period variables (Supplementary Data 5a), while no significant *p*-values were obtained for the other variables considered, thus excluding the effect of possible bias related to the sample processing process or to the

number of reads. We also performed a pairwise comparison between clusters, computing a pairwise ANOSIM and obtaining a significant result for almost all the comparisons (Supplementary Data 5b).
To explore which of the available bioarchaeological metadata were at the basis of the cluster subdivision, we created a classification model using random forest analysis, and found that the most important variables (VIPs) were the Period and Site. We validated the association between the Period variable with Cluster classification, performing an association test and computing Pearson X^2 (Supplementary Figure 8). We obtained a significant association between C1 and the Palaeolithic period, C2 with both the EN and MN periods, C4 with LN and CA period, while a sample belonging to the FMN (Palagiano site) was associated with two different clusters (C3 and C5), thus indicating a possible substructure within the same site.
By applying the DESeq2 comparison between pairs of clusters, we obtained several significant species that changed their relative abundances (Supplementary Data 6). To avoid spurious results, we kept only species that resulted as being significant (p adjusted < 0.05) from more than one comparison. Moreover, we created a generalised linear model using MaAslin2 between the microbiome species and the Period and Clusters variables, obtaining 31 significant associations from the model (Supplementary Figure 9 and Supplementary Data 7)

Olsenella sp. oral taxon 807 genome reconstruction

Finally, we performed a *de novo* reconstruction of an ancient oral bacterial genome following the pipelines in Wibowo *et al*.[38], selecting only good quality genomes (completeness >85%; contamination < 5%; taxonomic identification > 95%) and checking for contigs deamination profiles to confirm the antiquity of the assembled genomes (Supplementary Data 9 and Supplementary Figure 13). This approach enabled us to obtain different MAGs assembled, among which six genomes were identified as *Olsenella sp. oral taxon 807* by Kraken2. The taxonomic analysis conducted on our dataset highlights this species as one of the main markers of ancient farmer communities, in accordance with what was observed in another study for South Europe[35]. In order to analyse the evolutionary processes for this species, we compared reconstructed with modern genomes (Figure 5A). As expected, the phylogenetic analysis indicated the ancient Olsenella genomes to be closely related with the modern reference for this species but split into two different clusters: one composed of four genomes reconstructed from the most ancient samples, and two from CA samples that were phylogenetically closer to the modern reference. As is observable in Figure 5B, and presented in the main text, some regions were commonly missed among all the ancient genomes, which could be related to more recent elements of evolution. We controlled for the possibility that these regions may be missing due to degradation events related to their antiquity. To exclude the possibility of mapping bias related to GC content, we checked for the percentage of GC bases (%GC) and we observed that the mean value for the missing regions was close to that of the whole *Olsenella sp.* genome (0.65 and 0.62 respectively). Overall, a total of 123 missing regions have been detected from the genome comparisons, some of which are very small (such as <100 bp), while a subset of regions are fairly large (>1000 bp). Most portions of these regions contain only hypothetical proteins, while several contain sequences that are classified as protein families that appear to be depleted in ancient genomes (Supplementary Figure 14 and Supplementary Data 10). Two large gaps, located at ~1900kbp and ~2200kbp contains protein families that are involved in defence mechanisms and interactions with other microbial commensals, such as, among others, CRISPR-associated protein, mobile element, Fic domain proteins, integrase/recombinase, and tetracycline resistance family (TetR). We observed an absence of the CRISPR-associated proteins Csd1, Csd2, Cas2, Cas4 and Cas5, which are known to be part of the prokaryotic defence system against foreign genetic mobile elements such as those from viruses or transposable elements[39,40]. Antitoxin HigA is a part of the toxin/antitoxin system HigB/HigA has been proven to control virulence factor production in *P.aeruginosa* and biofilm formation[41]. Other missing proteins were those associated with filamentation induced by cAMP (Fic domain), which are involved in cell division and which are associated with bacterial virulence and biofilm formation[42,43]. Among the members of the TetR family, which appear to be absent in ancient genomes, we noted the transcriptional regulator LysR, AcrR and AraC families, which are involved in the expression of multidrug resistance genes[44,45].
Finally, a large visible depleted region at ~2800kbp was observable and it corresponded to dTDP-4-amino-4,6-dideoxygalactose transaminase and dTDP-4-dehydrorhamnose reductase, which is involved in outer membrane formation [46,47]. All these elements may account for a difference in ancient *Olsenella sp. oral taxon 807* in interacting with other microbial species that constitute the oral ecosystem and with the human host.

Results of microremains analysis in the upper Palaeolithic samples.
Plant micro-remains analysis was performed on the pellet residues from the dental calculus of nine individuals found at Paglicci. The number of remains, which mainly consisted of starch grains, was very different from sample to sample and independent of the weight of the samples (Supplementary Data 8).
Starch grains - A total of 215 starch grains was recovered in the samples. The majority of the starch grains displayed damages of different extent, from hardly visible to very serious. The damages are attributable to

food processing before eating[48–50] rather than to mastication that does not seem to appreciably alter the starch grain morphology[48]. Except for the unidentifiable, the starch grains were divided into morphotypes:
Morphotype I – Circular, sub-oval to oval grains in plain view (2D shape), with centric hilum. These grains are divided into two groups.
Morphotype Ia includes the grains with a diameter/main axis ranging from 10 to 47.5 µm. They are lenticular in 3D, the most part circular in 2D. The hilum is well marked, centric, and more or less dimpled; a linear fissure was rarely observed. Lamellae are visible, often evident; small dimples occur on the surface of some of the grains. Extinction cross is faded at various degrees. The majority of these grains share strong similarities with the grains of Poaceae, in particular with the type A starch grains of the Triticeae.
Morphotype Ib includes grains which have a diameter/main axis <10 µm, ranging from 3.5 to 9.5 µm. Some of them present dimpled hilum. The extinction cross is symmetrical, sometimes distorted at the center of the grains in correspondence with the dimpled hilum. They generally present slight damages. Their morphological features are not sufficient for their attribution to a group of plants.
Morphotype II - Irregularly ovoid starch grains with stellate, nearly centric hilum, the main axis measuring 20-23 µm. The extinction cross is bilaterally symmetrical. The shape of the grains is similar to that of the starch grains belonging to Fabaceae, *e.g. Vicia* (vetch). Anyway, they lack the typical longitudinal cleft fissure that is common in those grains. This morphotype was found in the sample PA25 only.
Morphotype III – Polyhedral starch grains, referable to compound starch grains: actually, four groups of a few of these grains were found, one in sample 133 and three in sample PA157.
The majority (IIIa) are irregularly polygonal grains in plain view, with rounded vertices, centric hilum surrounded by radiating fissures and generally symmetrical extinction cross. They measure from 10 to 20 µm. This morphology occurs in numerous Poaceae, for instance in Panicoideae and Polygonaceae; these grains are commonly attributed to “millet”.
The other grains (IIIb), discontinuously present in the samples, have an irregular, very angular shape with facets irregularly concave; centric hilum with radiating fissures; extinction cross distorted by the facets. The main axis ranges from 12 to 25 µm, exceptionally 28 µm. They presented a good state of preservation. This type of starch grains was found on the surface of a Gravettian pestle-grinder from Paglicci and attributed to *Avena* cf. *barbata* (oat, cf. slender wild oat).
Morphotype IV - Irregularly ovoid starch grains with very eccentric hilum, the main axis measuring 44.5-50 µm. The extinction cross is asymmetrical with curving arms. The grains are strongly damaged. They have the same morphology of the grains stored in the rhizome of *Nuphar lutea* (yellow water lily).
Two groups of grains with bimodal size distribution were found in the samples: one in sample PAB 14 B and one in sample PA 150. The grains, especially the large ones (type A), are strongly damaged. They exhibit centric hilum, well visible lamellas and faded extinction cross. The large grains are circular, 39-43 µm and 25 µm in diameter; the small grains (type B) range from 9.8 and 5.4 µm in diameter. They may be attributed to Triticeae, the local wild relatives of the Middle East domesticated species. Direct evidences are reported on Supplementary Figure 16.

Results of microremains analysis in Neolithic samples.
Archaeological dental calculus and pellets preserved various vegetal and animal micro-debris (Supplementary Data 8). Overall, dental calculus and pellet samples yielded comparable plant and animal micro-remains, in the form of starch grains, phytoliths, plant fibers, plant parenchyma, fungal spores, diatoms. Few and tiny charcoal fragments and vegetal tissues were also retrieved in the matrix. However, based on their dimensions, frequency, and typology, it was impossible to formulate any identification or hypothesis about their presence in the matrix.
Starch grains - Starch grains were recovered in the pellet residue of 3 out of the 7 individuals analysed and in the dental calculus of 6 out of the 13 individuals analysed (Supplementary Data 8). Overall, 2 morphotypes have been retrieved in this study. In order to avoid misinterpretation grains smaller than 5 µm (transitory starches) were not considered[51].
Morphotype I. Morphotype I grains was the retrieved in both pellet and dental calculus samples. In particular, starch grains from this type were found in 3 individuals from the site of Masseria Candelaro (CND40_IDA; FFB4A; CND92), 2 individuals from Palagiano (Pala T3-23; Pala T3-no), and 2 individuals from Ripatetta (Rip.USD618; Rip.USD352). Some of the starch grains of this type were found still partly embedded within the calculus remains. Size, shape, morphology that characterize grains of this type are encountered in the members of the plant tribe Triticeae (Poaceae family) and considered diagnostic features for taxonomic identification[48,52,53]. Starch grains of this type recovered in the archaeological dental calculus and pellet involves the presence of large grains, round to sub-oval in 2D shape, ranging between 21.1 and 45,1 µm in maximum dimensions (mean size of 33,1 µm), lenticular 3D shape, a central hilum, a visible equatorial groove and deep lamellae concentrated in the mesial part; and small (< 10 µm) grains (B-Type) with round/sub-oval shapes and a central hilum [52,53]. Based on literature[48,53] and our modern reference collection, this characteristic is common in type A grains of the species of the Triticeae tribe[52,54].

Morphotype II. One starch granule of this type was identified in 1 individual from Masseria Candelaro (FFB4A). Starch grains of this type show a reniform shape in 3D, a collapsed/sunken hilum forming a deep fissure along almost the entire granule, a size ranging between 12 to 35 μm and a very bright extinction cross with several lateral arms diverging from the hilum[48]. All these features are very peculiar and diagnostic of starch grains included in the species of the plant family Fabaceae[48], known for several edible domesticated species of legumes (*e.g.,* lentil [*Lens culinaris* Medikus]*,* fava bean [*Vicia faba* L.], and pea [*Pisum sativum* L.]) and wild vetches (*Vicia* spp.). Identification at species or genus was not possible due to overlaps in shape and size of starch grains at tribe level, which were observe d in our modern reference collection.

Diatomee - Several comparable diatoms were recovered entrapped in the calculus matrix of an individual from Masseria Candelaro and Ripatetta. Two different species of diatoms were identified. In the dental calculus of individual CND40_IDA, diatoms of the genus Nitzschia have been identified. Nitzschia genus is relatively large and includes many freshwater and marine species, generally pollution-tolerant. The diatoms recovered in our archaeological samples are more likely associated with freshwater based on their morphology and dimensions. Many species of the Nitzschia genus are epipelic and live in mud environments at the interface of the water and sediment. Their presence in the dental calculus of one individual from Masseria Candelaro can hence be considered indirect evidence of the use of polluted waters. Fragmentary of freshwater diatoms possibly belonging to the genus Surirella or Cymatopleura were also retrieved in the dental calculus more individuals from Ripatetta (Rip.USD360) and Masseria Candelaro (CND40_IDA).

Phytoliths. - Phytoliths were identified in all the samples analyzed for a total of 118 cells. Amongst them, it is worthy of mentioning the recovery of 17 elongate dendritic epidermal phytoliths. Within the pellet samples, they were found in 4 individuals from Masseria Candelaro (CND40_IDA; CND_Cr5; CND86; FFB4A; CND92) and 2 individuals from Portonuovo (US1056_I and US1056_II). Within the dental calculus, they were found in 1 individual from Masseria Candelaro (CND91). The retrieval of this type of phytoliths in dental calculus is generally considered direct evidence for cereal consumption as they produce in the inflorescence bracts of wheat and barley species [55].

Fungi- Fungal spores are well represented in the dental calculus of 7 individuals from Masseria Candelaro (CND91), Deliceto (DEL77), Palagiano (P.T10.1 and P.T3.21), and Ripatetta (Rip.USD613; Rip.USD697; Rip.USD360) and in the pellets extracted from the dental calculus of two individuals from Masseria Candelaro (CND_Cr5; FFB4A). At least three individuals (Rip.USD613; P.T10.1 and FFB4A) spores recovered in the dental calculus likely belong to Glomus, the largest genus of Mycorrhizal fungi growing in association with plant roots. Interestingly, in the dental calculus of one individual from Masseria Candelaro (FFB4A) and Ripatetta (Rip.USD613), several fragments of parenchyma tissue, abundant in the cortex of roots, were retrieved together with glomus fungal spores. Saccharomyces spores were also identified in two cases (P.T10.1; Rip.USD360). They are also known as “sugar fungi” and include several yeasts, suggesting food fermentation processes.

Animal debris - Together with vegetal material also one barbule was recovered in the dental calculus of one individual from Ripatetta (Rip.USD613). Based on some microscopic characters of the barbs, i.e. the morphology of the nodes observed at high magnification and our experimental record, we suggest the fragment of barbule may belong to an aquatic animal of the Anatidae family.

Other - Within plant micro-record few unidentifiable plant tissues, wood elements and fibres have been identified. Direct evidences are reported on Supplementary Figure 17.

Supplementary Figures

Supplementary Figure 1: Comparison with other microbiomes sources. PCoA draw on Bray-Curtis analysis between ancient samples from this study and other microbiome sources. It is possible to observe that the ancient samples produced in this study mainly clustered in proximity of other published ancient dental calculus microbiome (49 samples) and close to modern oral microbiomes (425 samples). Moreover, they are deeply separated from environmental and soil microbiomes (54 samples) and negative laboratory controls (2) as well as from other human microbiome sources, such as skin (23 samples) and stool (128 samples).

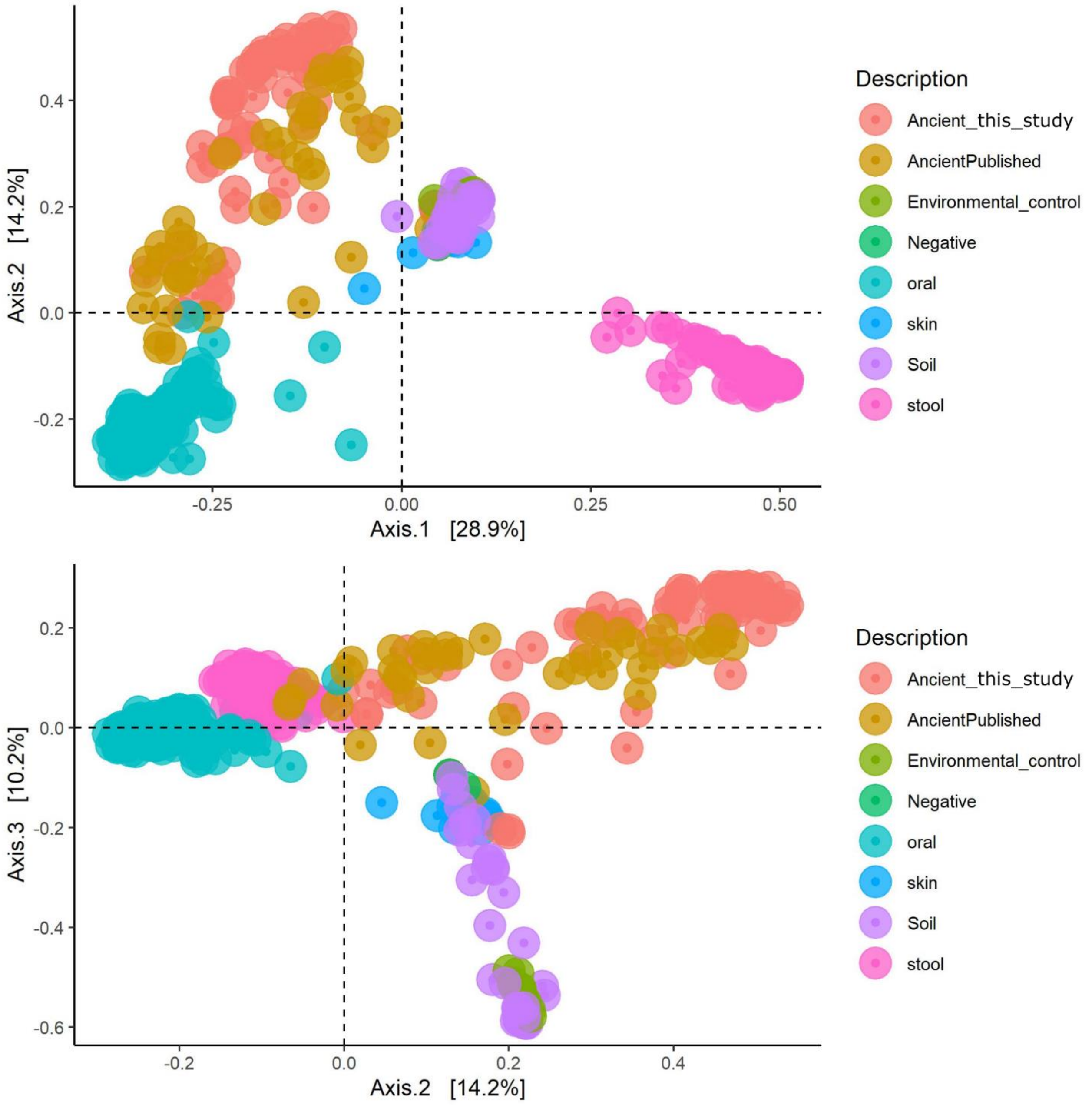

Supplementary Figure 2: Sourcetracker result. Different microbiome sources produced through shotgun approach using an Illumina platform and with >10 million reads, where used for the present analysis: stool (10), soil (20), modern dental calculus (10), subgingival plaque (15), skin (10), laboratory negative control (2). Most part of samples (71) demonstrated a genuine oral profile.

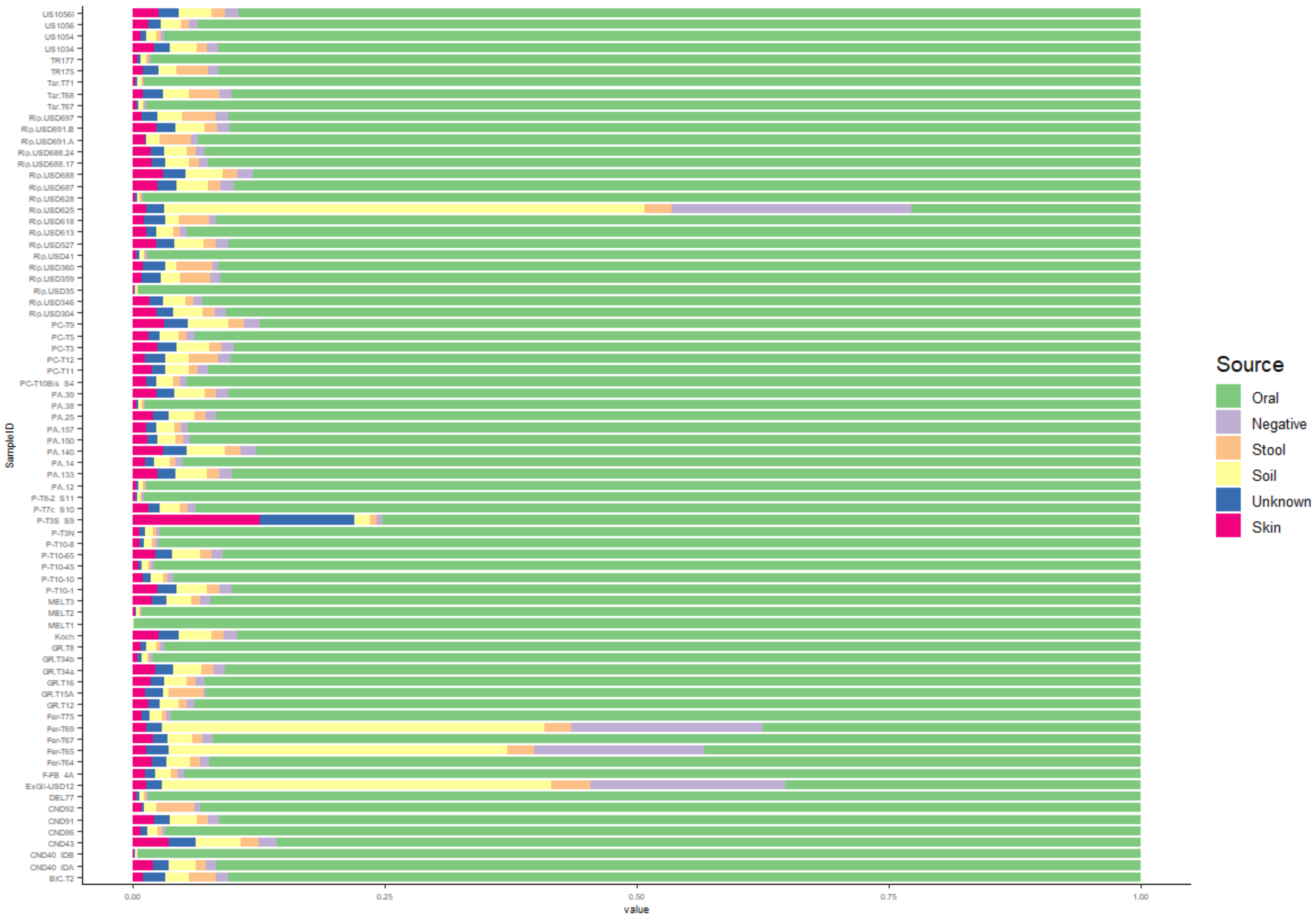

Supplementary figure 3: Representation of the deamination curve C to T for each one of the 49 species identified. The red line, reporting the deamination level, was obtained from the estimation of the mean value of C to T at each base in position 5’ from all the considered samples. Black lines represent the standard deviation.

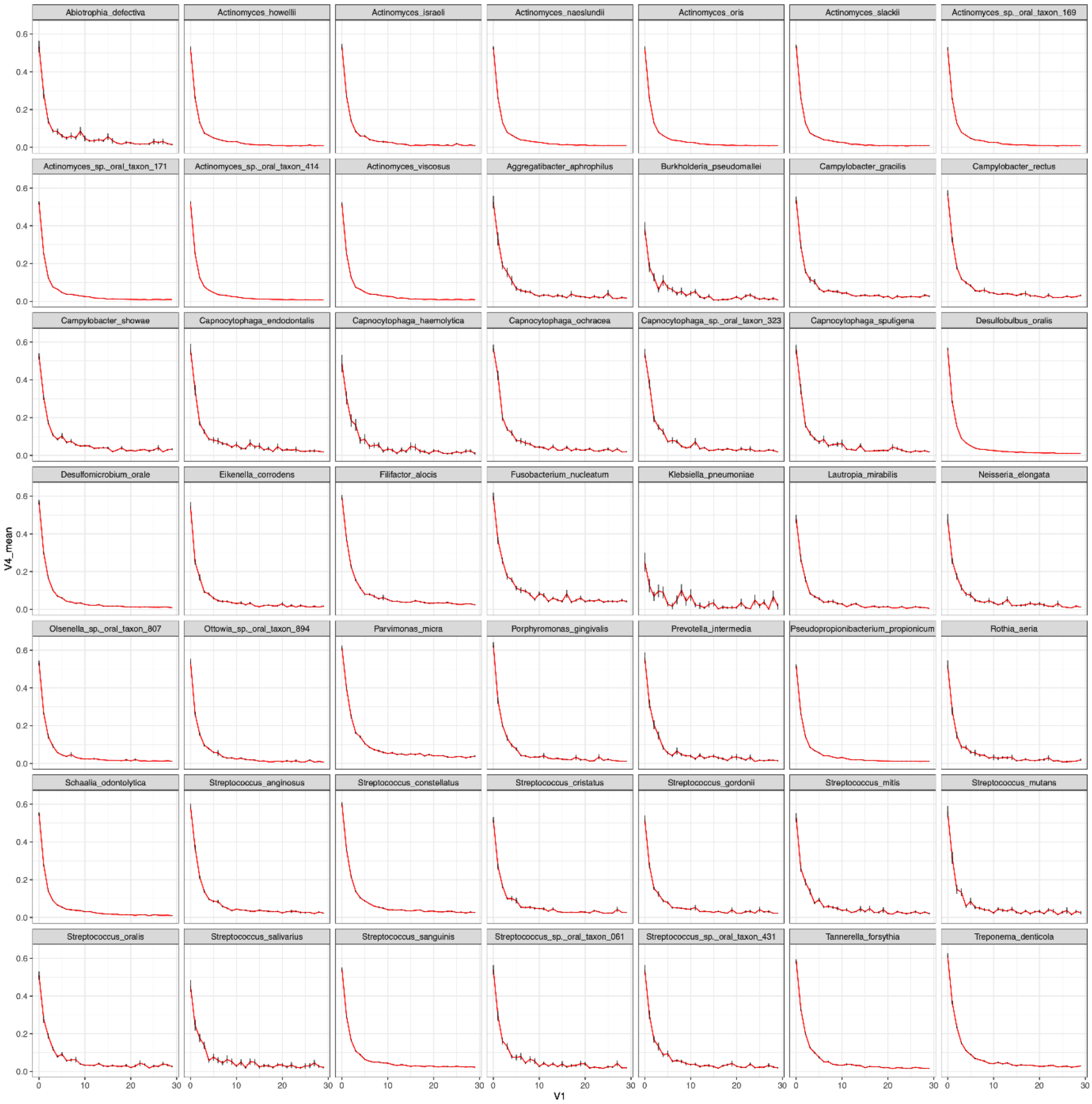

Supplementary Figure 4: Percentage of C-T 5' deamination. Boxplots represent the values of C-T 5' deamination % for samples (n=72) belonging to each period analysed. Wilcoxon test have been applied to highlight possible differences in deamination rates among different period but no significant values were identified.

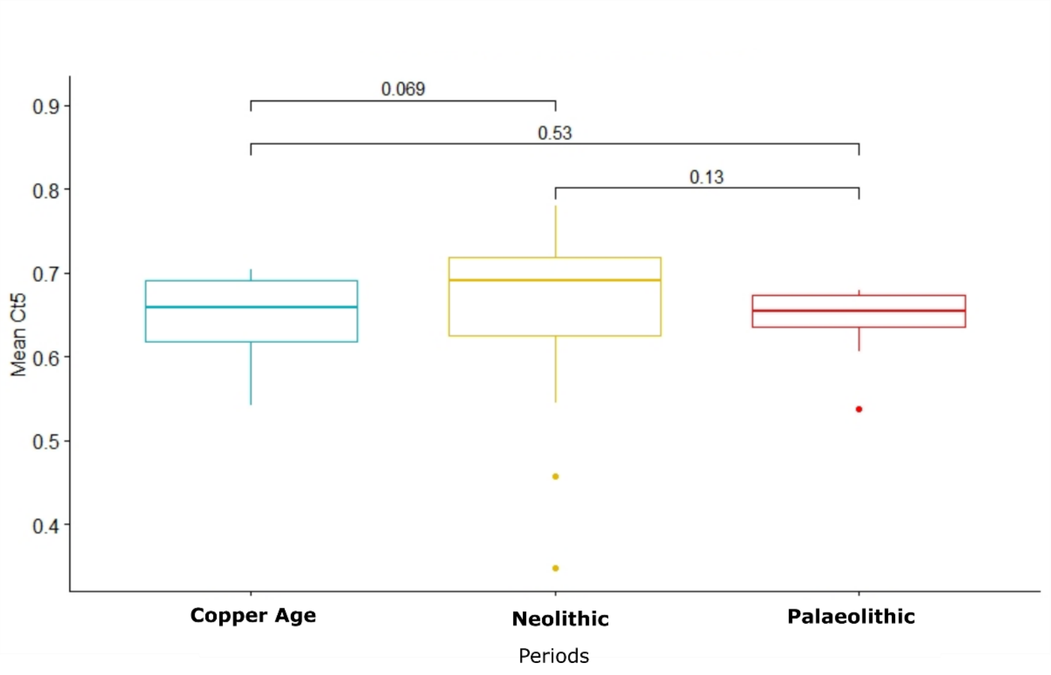

Supplementary figure 5: Barplot representing the number of reads for each fragment length range present in cleaned fastq. Each graph shows data for each period analysed: PA (A), EN (B), MN (C), FMN (D), LN (E), CA (F).

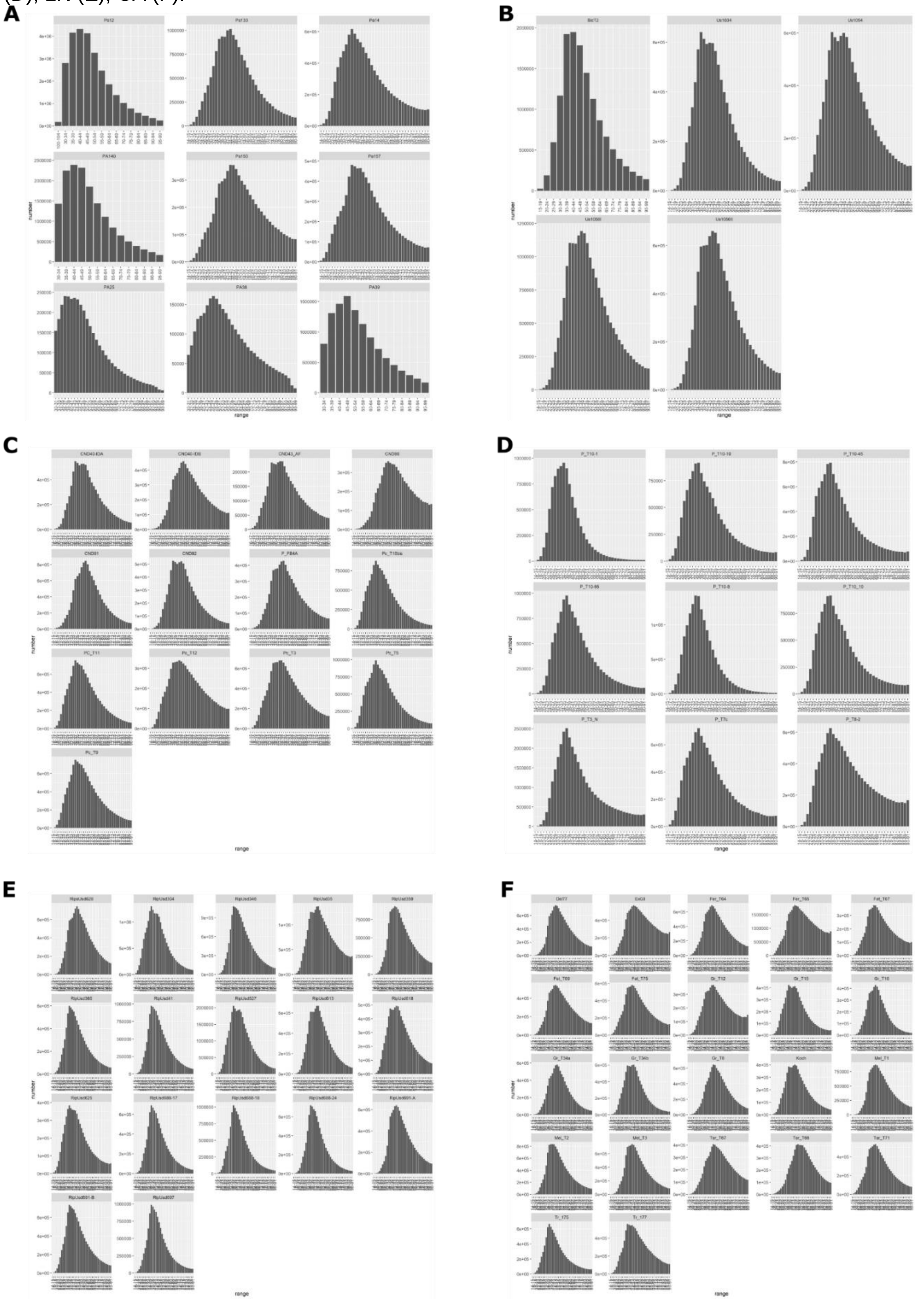

Supplementary Figure 6: PCoA on Aitchison distance. PcoA axis 1-2 (A) and axis 2-3 (B) are here reported. The substructures related to microbiome composition identified by network analysis are here represented. Clusters (C*n*) are identified by different colours, while Period variable is represented by different dot shapes. Colours are in accordance with Network clusters.

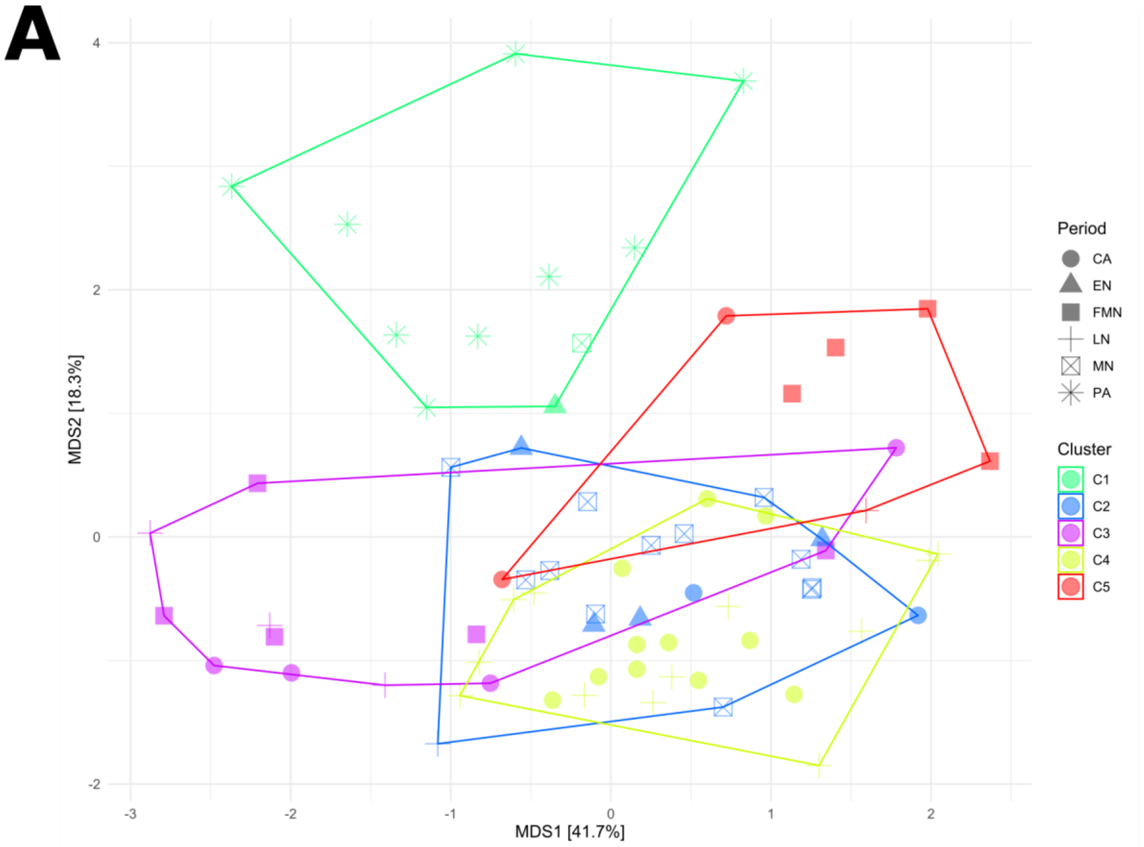

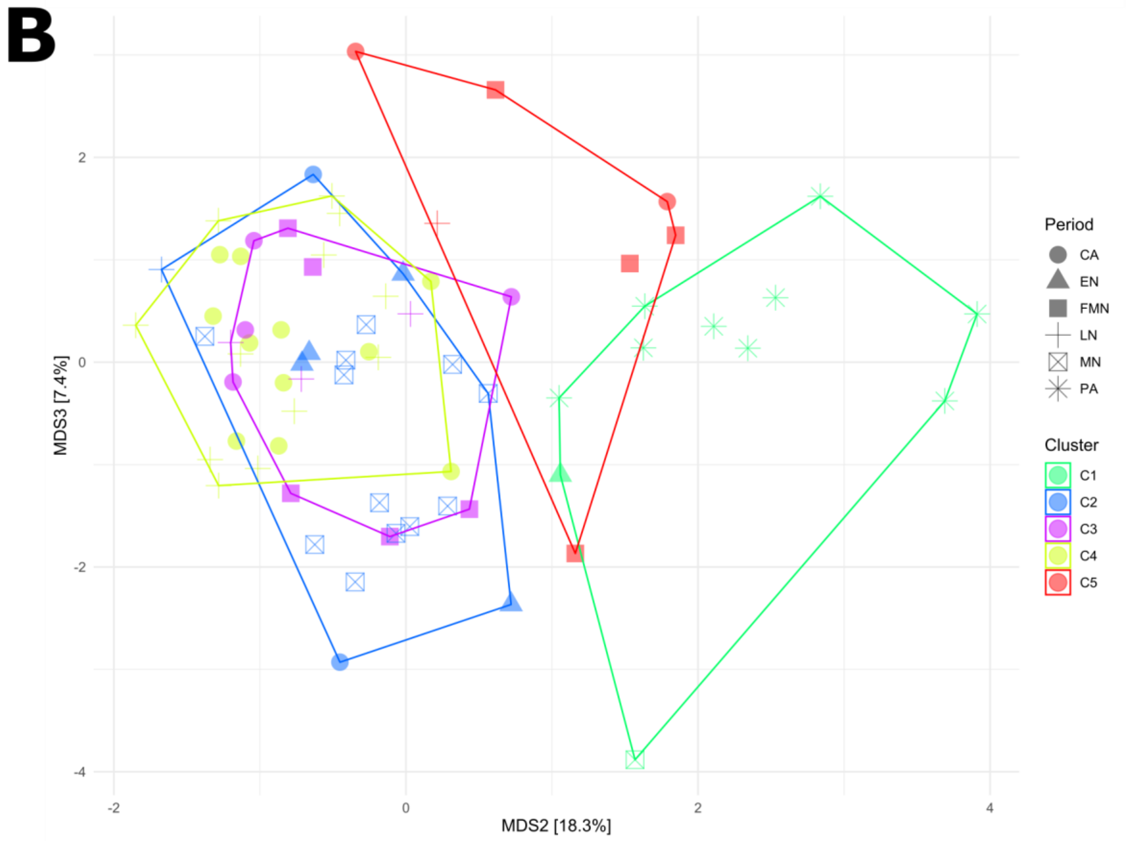

Supplementary Figure 7: PCoA analysis on sample processing variables. We tested if the obtained clusters could be possibly related to some variables associated to sample processing, such as: the number of reads after trimming used for taxonomic classification(A); the extraction batch (B); the library batch (C). No clusters were associable to any of these variables, avoiding their influence in the analysis (Supplementary Data 5).

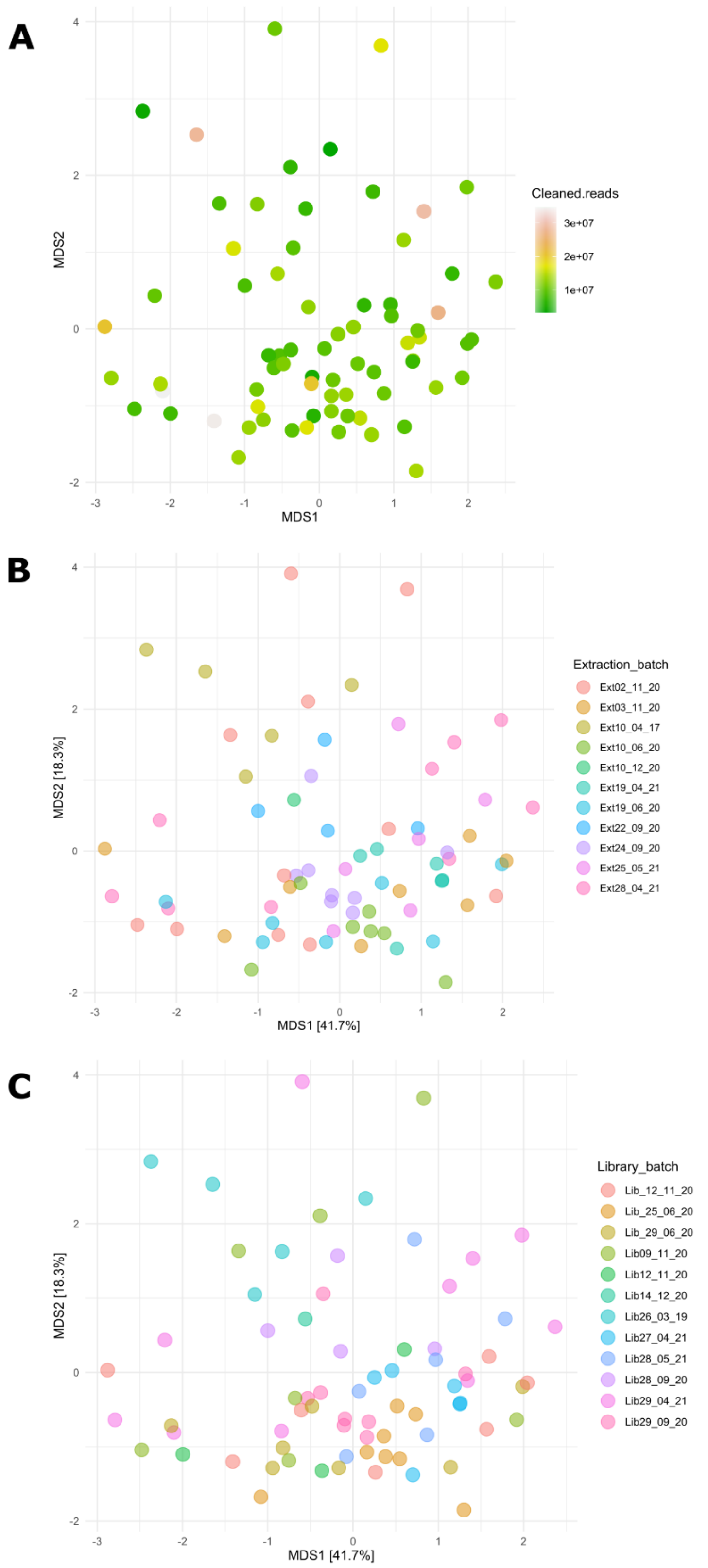

Supplementary Figure 8: Association test between period and clusters variables. Blue bar indicates positive association while red bar indicates negative one.

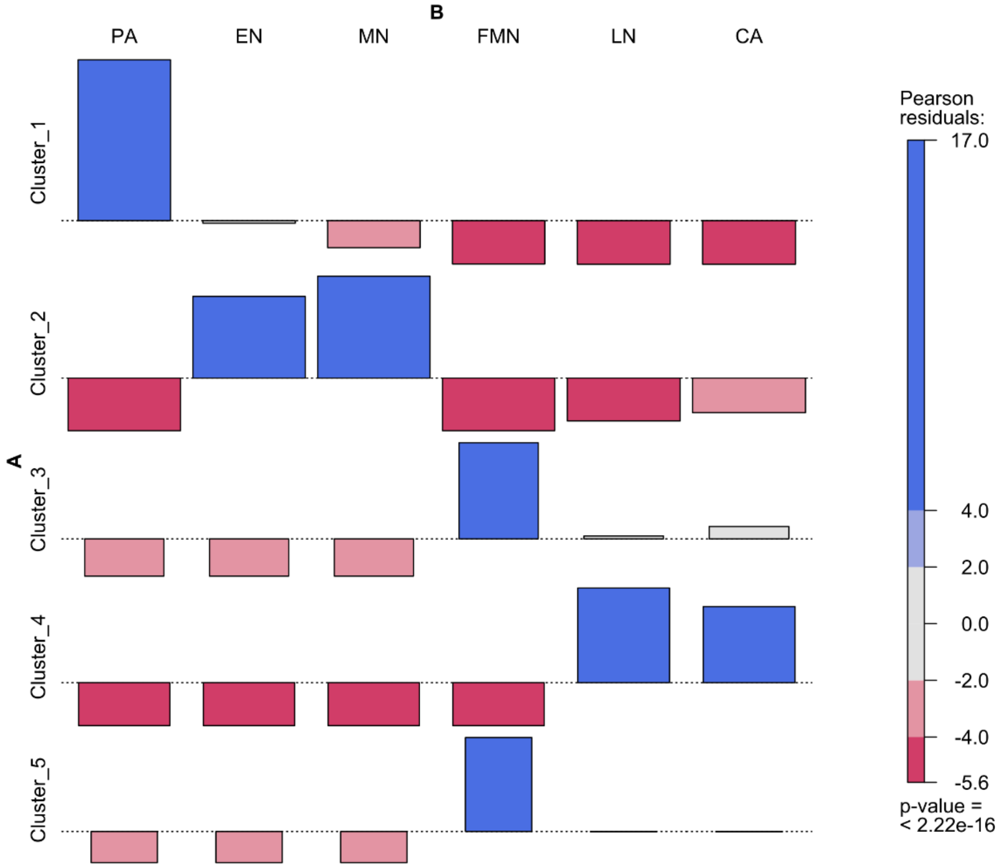

Supplementary Figure 9: Results from Multivariate Analysis by Linear Models (MaAsLin). We obtained that 16 species were differently associated with Clusters (A) or Period (B). Negative/Positive coefficient values (x-axis) represent the type of association between species and metadata. Three species were positively associated with PA period, further highlighting microbiome specificity associated with hunter-gatherers' community with respect to the Neolithic samples. Different association were found among the clusters that characterize the Neolithic period. Some of them are shared across all the Neolithic clusters (such as *Streptococcus gordonii*, *Streptococcus mitis*, *Streptococcus sp. oral taxon 061*, *Aggregatibacter aphrophilus* and *Abiotropia defectiva*), suggesting that their negative association is common for all the Neolithic samples. Other associations are instead characteristic of only some specific clusters, such as *Porphyromonas gingivalis* and *Desulfomicrobium orale* with C4 (LN/CA period). Source data are provided as Source Data file.

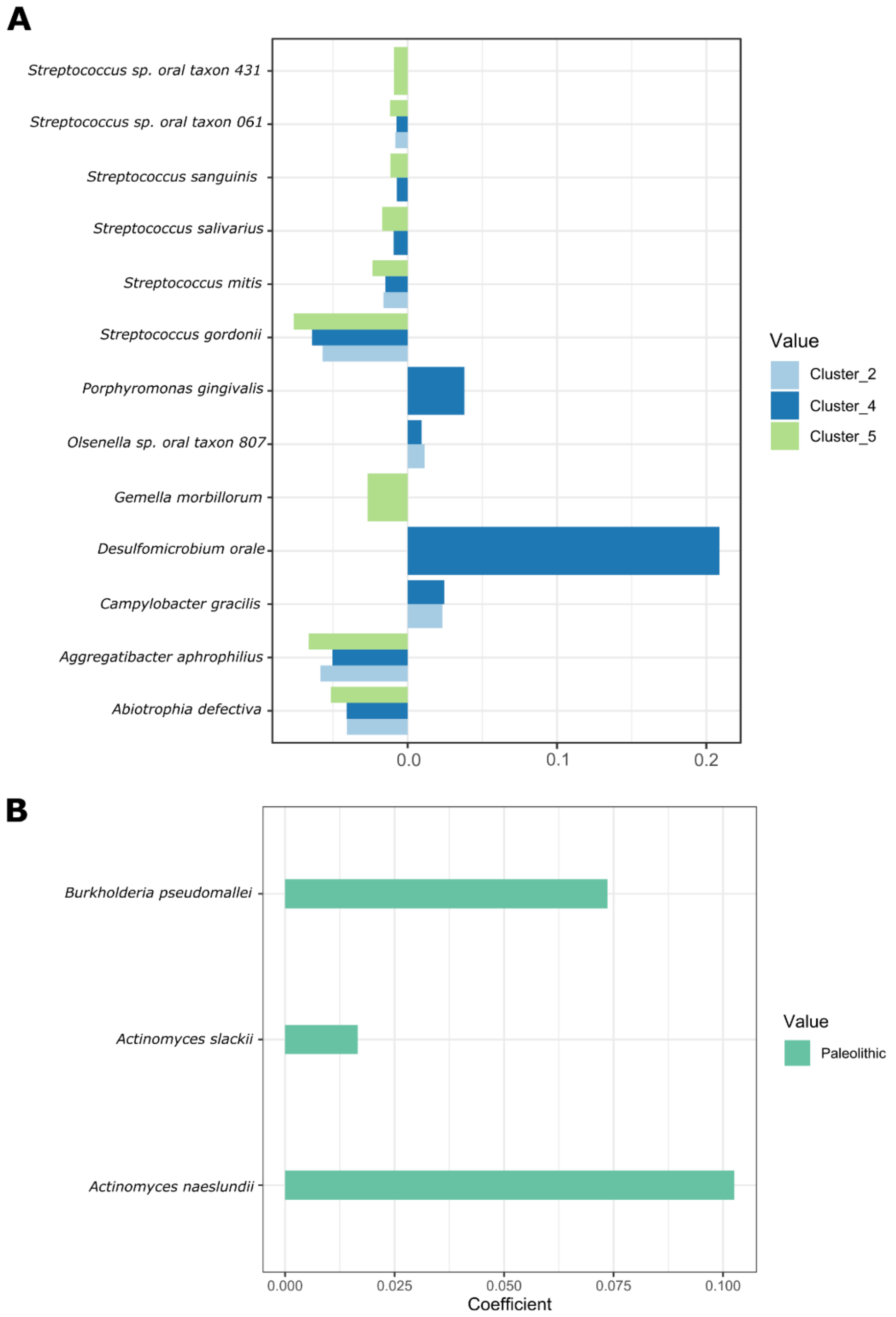

Supplementary Figure 10: Significant species out of major oral complexes. Significant microbiome species obtained through DESeq2 test and not directly associated to one of the major ecological complexes that characterize the oral microbiome. Samples (n=71) are divided by period on the x-axis, while species relative abundance is reported on y-axis. Source data are provided as Source Data file. Boxplots are defined as follow: minimum, maximum and centre values represent the 25%, 75% and 50% quantile, respectively; median is represented by 50% quantile; upper and lower whiskers are the maximum/minimum values of data which are within the 1.5 interquartile range over 75th or under 25th percentile, respectively.

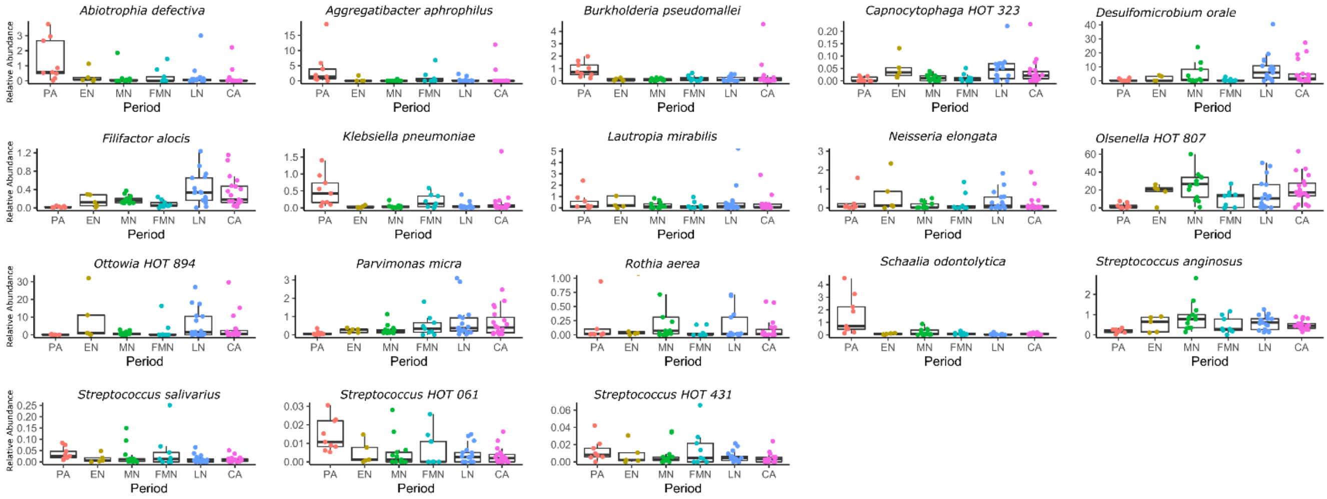

Supplementary Figure 11: Results from the analysis of LEfSe on gene abundance across the identified clusters. Cluster 1 and 4 are the one with more significant differences and correspond to the LN/CA and PA samples respectively.

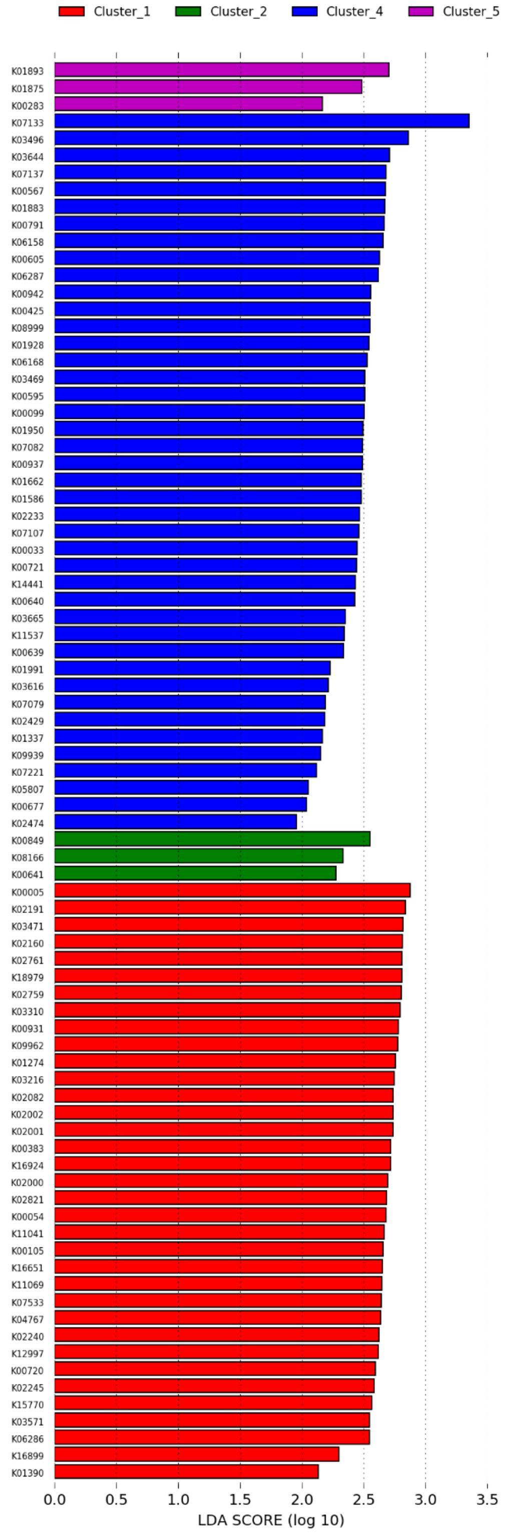

Supplementary Figure 12: Glycan and Vitamins KEGG Orthologs. Panel A and B reported the significant KEEG orthologs identified by LEfSe associated to glycan and vitamin pathways, respectively. For both panels samples (n=71) are grouped by period on the x-axis, and KEEG orthologs relative abundance is reported on y-axis. Source data are provided as Source Data file. Boxplots are defined as follow: minimum, maximum and centre values represent the 25%, 75% and 50% quantile, respectively; median is represented by 50% quantile; upper and lower whiskers are the maximum/minimum values of data which are within the 1.5 interquartile range over 75th or under 25th percentile, respectively

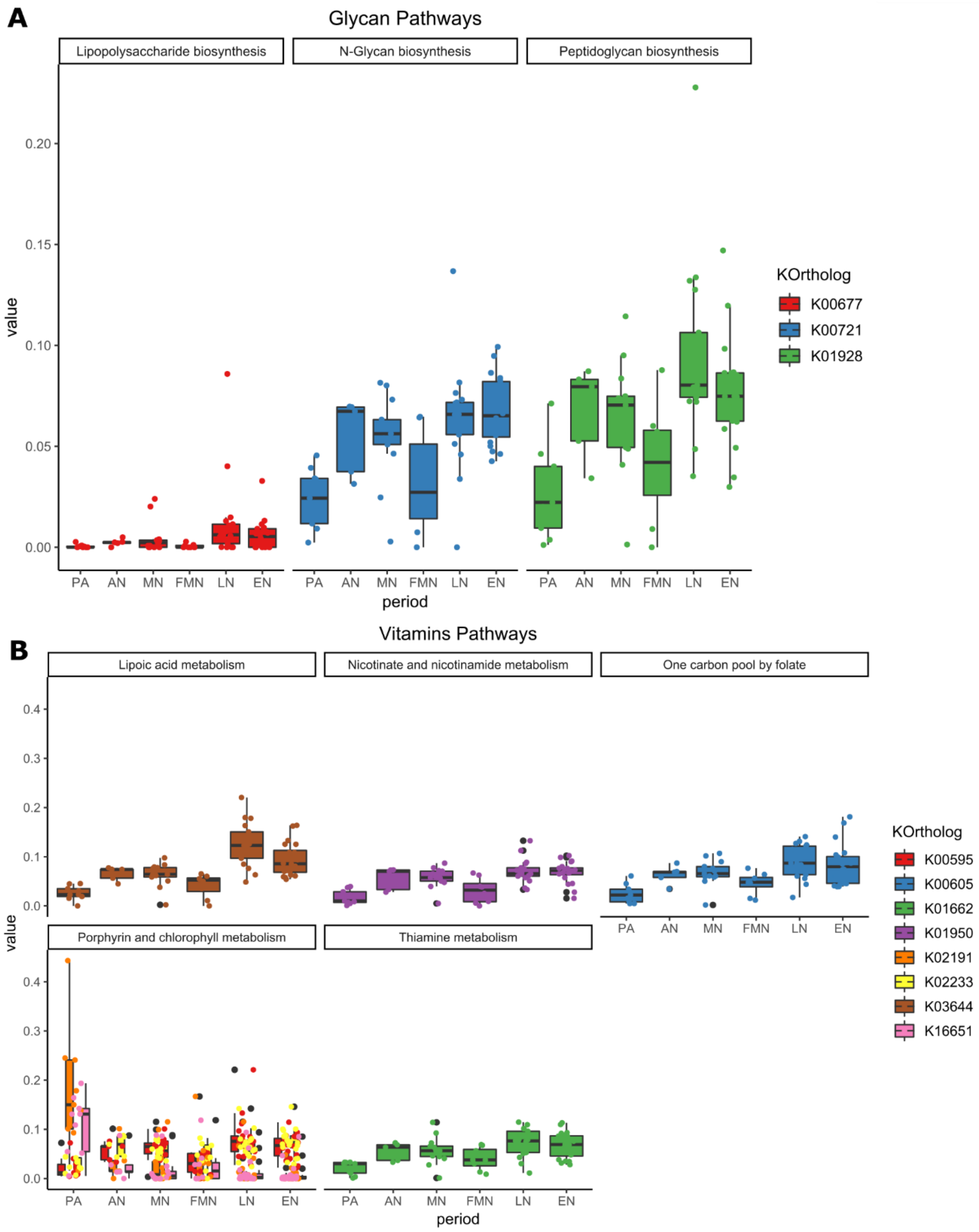

Supplementary Figure 13: Plot of ancient DNA damage pattern for all the Olsenella sp. oral taxon 807 contigs obtained for each sample. The analysis was conducted following Wibowo et al.(43) damageprofiler.sh script. In summary: each sample is aligned against all the contigs generated using bowtie2 in order to generate a SAM file. Then only aligned reads are used to generate the BAM file (-bSF4 -@ 30) which is subsequently indexed and sorted. Finally, all the rows with <1000 reads are removed and analysed for their damage pattern using PMDtools.

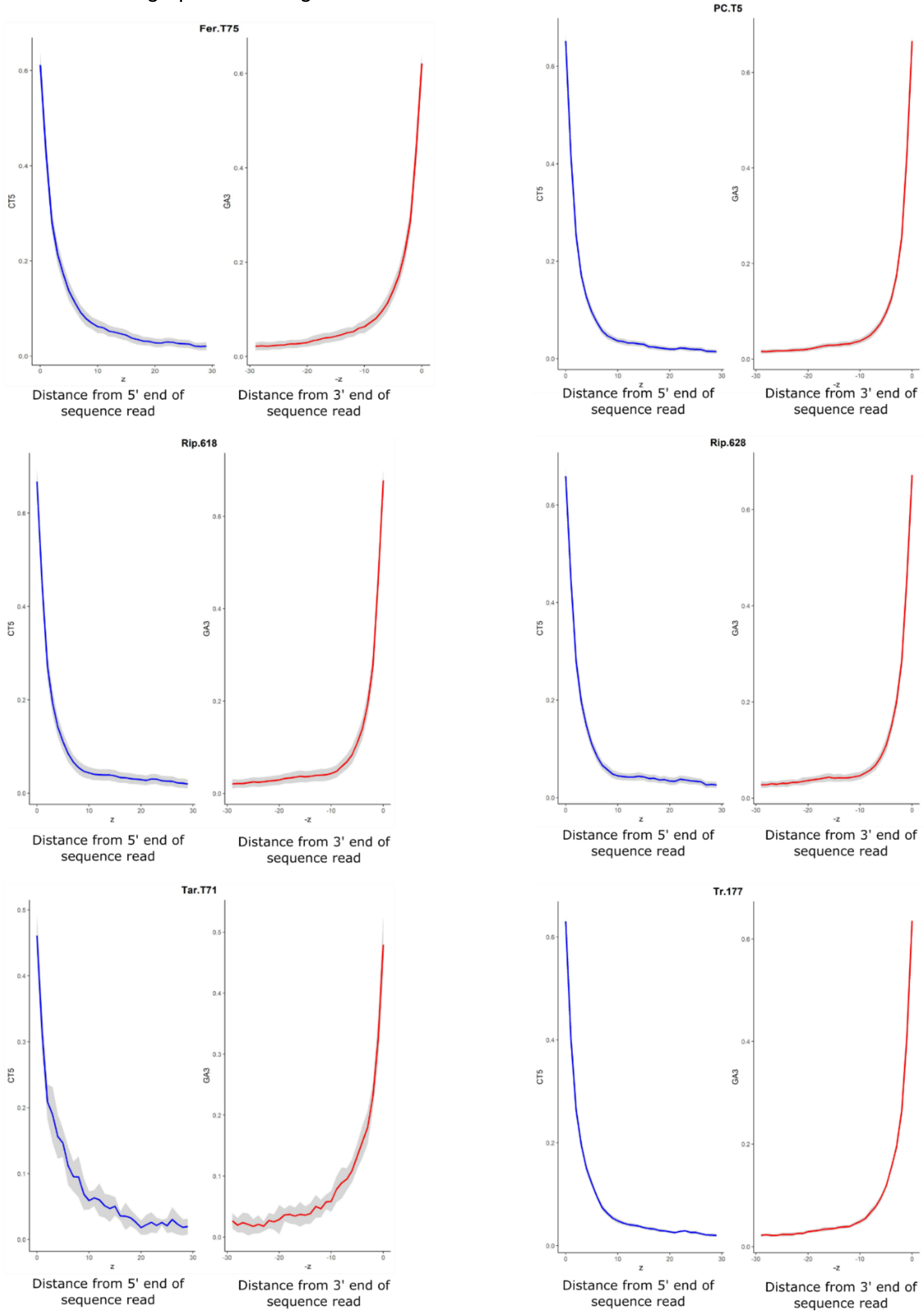

Supplementary Figure 14: Olsenella missing regions. Alignment analysis conducted using BRIG[56]. Red labels report the genes or protein products associated to the missing regions in all the ancient genomes.

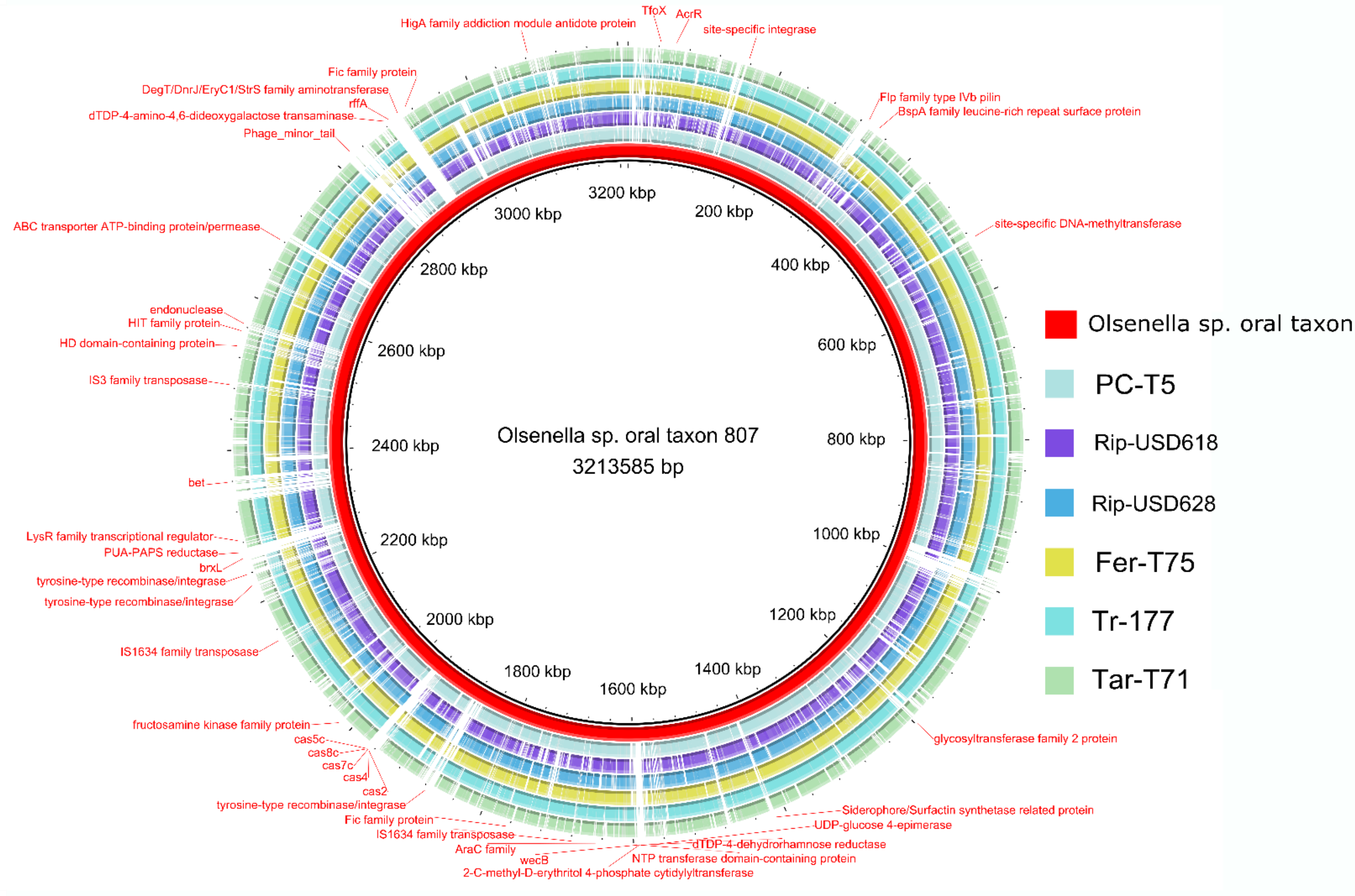

Supplementary Figure 15: Correspondence between chronology and paleoclimate data. Correspondence of the investigated time (i.e., chronology), with the paleoclimate reconstruction (*i.e.*, Climate) for the Neolithic period in South-Italy derived by Fiorentino et al.[21], the cultural background (third block), the selected archaeological site (fourth block) and the results from the cluster analysis (C1-C5) reported in Figure 2 Panel A in the main text. In the “Cultural background” block, each Neolithic culture is defined by a different colour, which highlights its association to the archaeological sites (third block) and to the specific Neolithic phase reported in the headings of the “Clusters” block (i.e, EN, MD, FMN, LN).In the last block (i.e., called “Clusters”), samples numbers are reported on the x-axis and cluster annotation on the y-axis. Each bars’ colour is associated to clusters ‘colours reported in Figure 2 Panel A. Within each bar, the percentage of samples falling within a specific cluster is reported in bold. Climate block is retrieved by Fiorentino et al. data and the dark grey bands highlight the two identified dry phases. Interestingly, the beginning of dry phases coincides with the beginning of LN period.

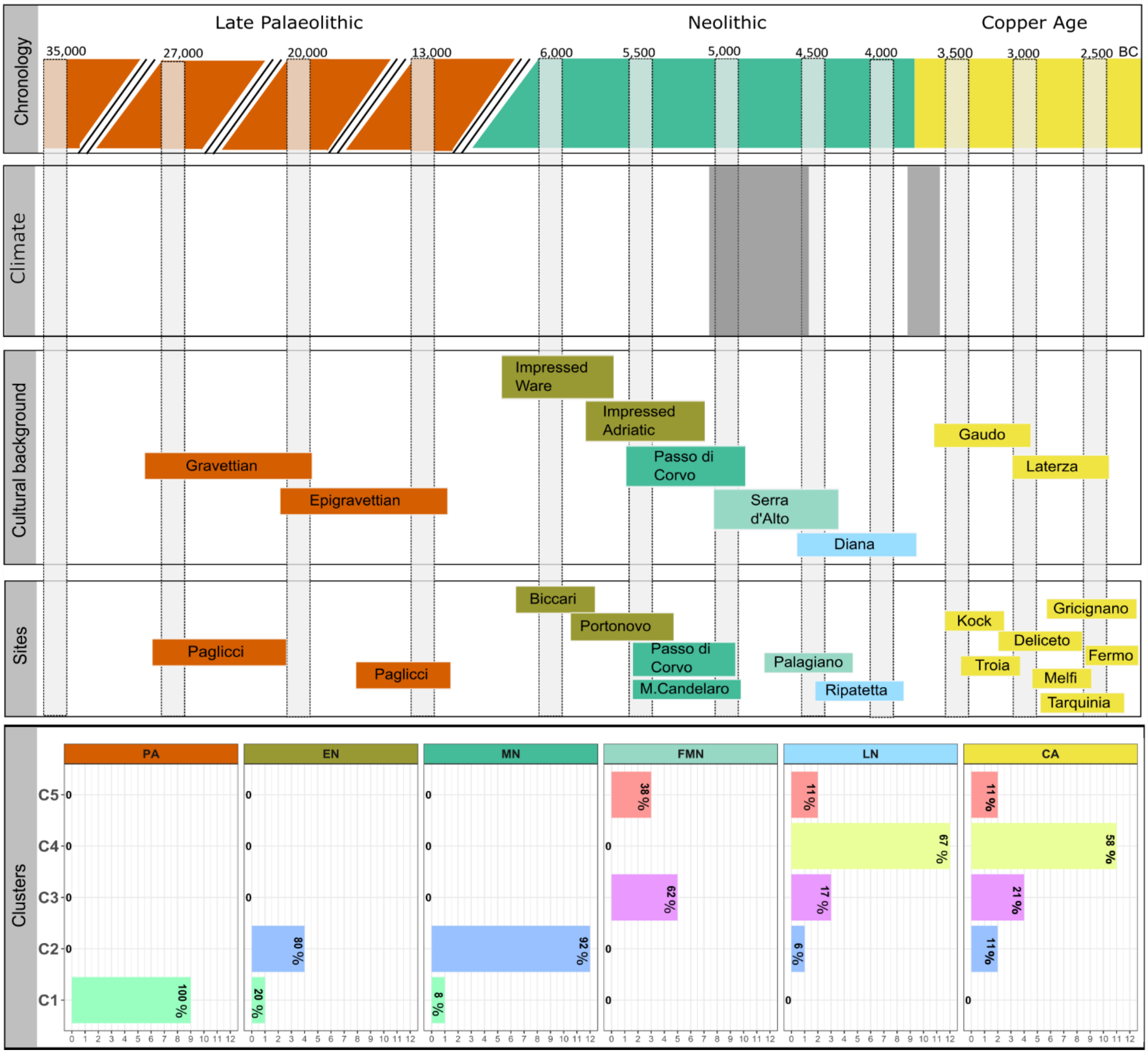

Supplementary Figure 16: Palaeolithic micro-debris results. A) Morphotype Ia in sample PA14B; B and C) Morphotypes Ib in sample PA157 and PA14 B, respectively; D) Morphotype IIa in sample PA157; E) Morphotype IIb in sample PA157; F) Morphotype III in sample PA25; G) Group of starch grains with bimodal size distribution PA14 B; H) Morphotype IV in sample PA14 B; I) Modern example of *Nuphar lutea* from the botanical garden of the University of Florence

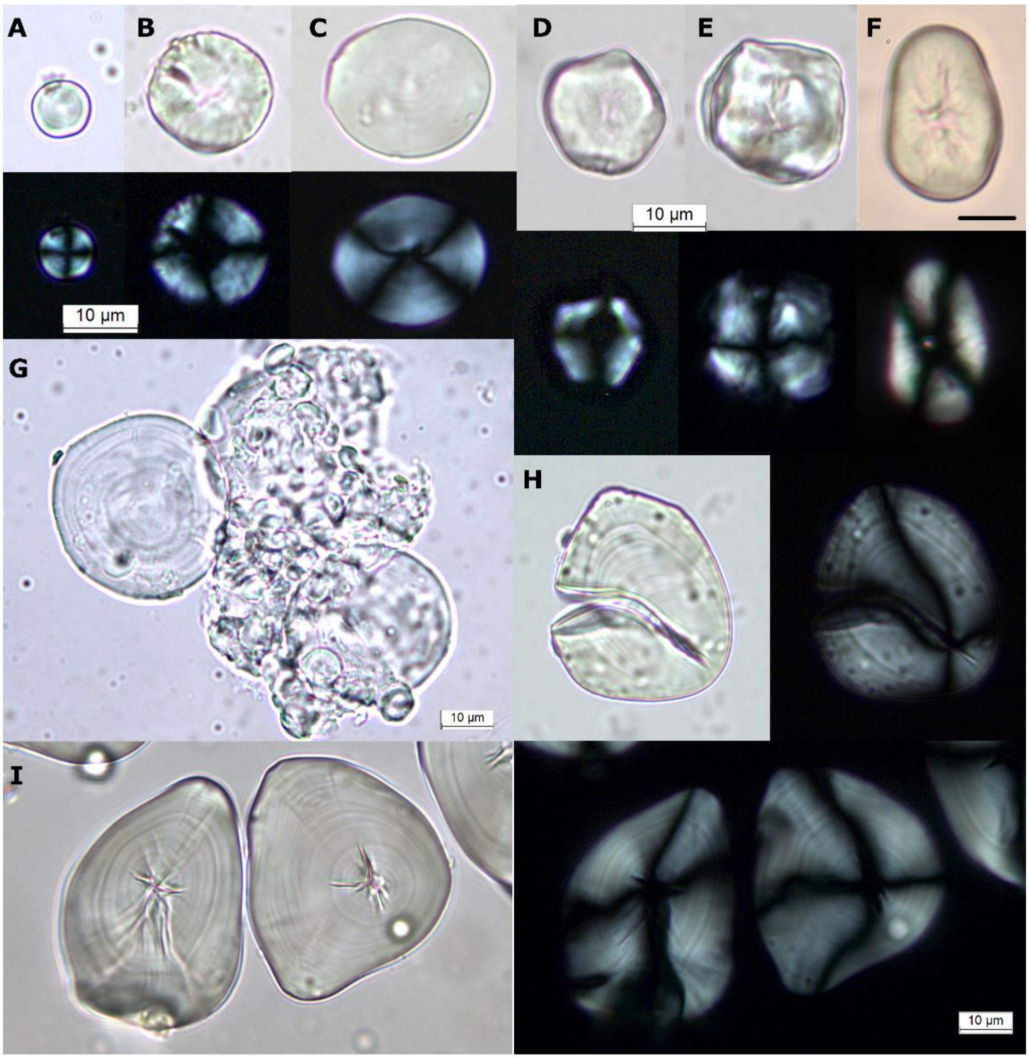

Supplementary Figure 17: Neolithic and Copper Age micro-debris results. A and B) Morphotype I from sample P.T3N; C) Morphotype I from sample DEL77; D, E and G) Diatomee from samples CND92, CND40_IDA and CND86, respectively; F and H) Phytolitis from samples CND40_IDA and CND_CR5, respectively; I, L, M and N) Fungal spores from samples FFB4A, Rip.USD360, Rip.USD613, CND91, respectively; O) Fragment of barbule probably from aquatic animal of the Anatidae family found in sample Rip.USD613.

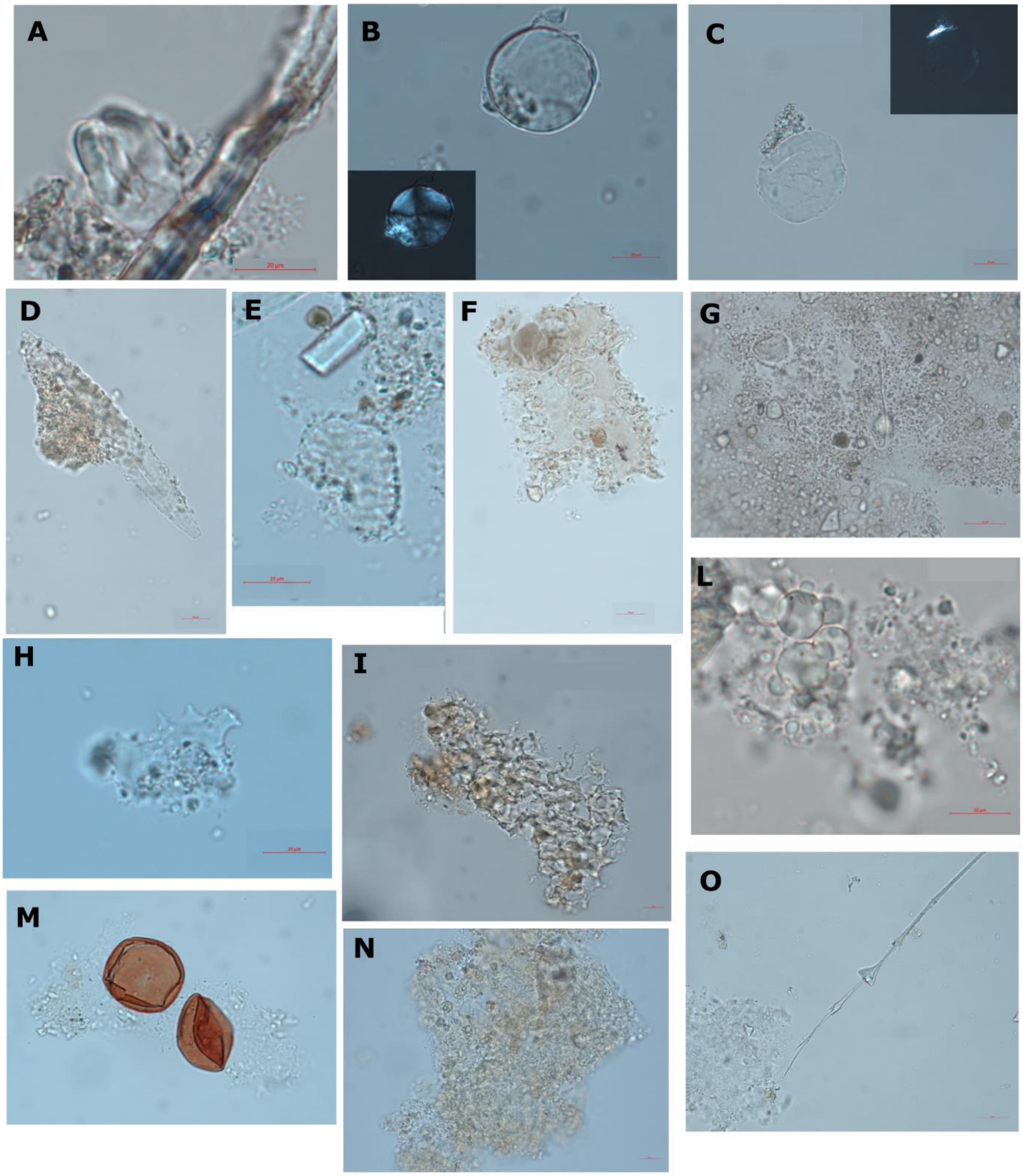

Supplementary References

1. Berto, C., Boscato, P., Boschin, F., Luzi, E. & Ronchitelli, A. Paleoenvironmental and paleoclimatic context during the Upper Palaeolithic (late Upper Pleistocene) in the Italian Peninsula. The small mammal record from Grotta Paglicci (Rignano Garganico, Foggia, Southern Italy). *Quaternary Science Reviews* **C**, 30–41 (2017).
2. Palma di Cesnola, A. *Le paléolithique supérieur en Italie*. (J. Millon, 2001).
3. *Paglicci: l'Aurignaziano e il Gravettiano antico*. (C. Grenzi, 2004).
4. Giaccio, B. *et al.* The Campanian Ignimbrite and Codola tephra layers: Two temporal/stratigraphic markers for the Early Upper Palaeolithic in southern Italy and eastern Europe. *Journal of Volcanology and Geothermal Research* **177**, 208–226 (2008).
5. Mezzena, F. & Cesnola, A. P. D. Scoperta di una sepoltura gravettiana nella Grotta Paglicci (Rignano Garganico). *Rivista di scienze preistoriche* 27–50 (1972).
6. Mezzena, F. & Palma Cesnola, A. Nuova sepoltura gravettiana nella Grotta Paglicci (Promontorio del Gargano). *Rivista di Scienze Preistoriche* **XLII**, 3–29 (1989).
7. Ronchitelli, A. *et al.* When technology joins symbolic behaviour: The Gravettian burials at Grotta Paglicci (Rignano Garganico – Foggia – Southern Italy). *Quaternary International* **359–360**, 423–441 (2015).
8. Reimer, P. J. *et al.* The IntCal20 Northern Hemisphere Radiocarbon Age Calibration Curve (0–55 cal kBP). *Radiocarbon* **62**, 725–757 (2020).
9. Ramsey, C. B. Dealing with Outliers and Offsets in Radiocarbon Dating. *Radiocarbon* **51**, 1023–1045 (2009).
10. Ricci, S. *et al.* Toothpick use among Epigravettian Humans from Grotta Paglicci (Italy). *International Journal of Osteoarchaeology* **26**, 281–289 (2016).
11. Lieto, M. M. D. L. D. A.M. Tunzi, M. Lo Zupone, M. Di Lieto, L'insediamento neolitico stagionale di Serra di Cristo (Biccari - FG), in A. Gravina, (a cura di) Atti del 34° Convegno

nazionale "Preistoria, Protostoria e Storia della Daunia" San Severo (FG), 16-17 Novembre 2013, San Severo 2014, pp. 53-80. (2014).

12. Tunzi, A. M. & Lozupone, M. Biccari (Foggia) – Storie di “abitati minori” del Neolitico: la tomba della “signora” e l’atelier delle “veneri”. in 129–149.
13. Conati Barbaro, C. *Il fuoco e la memoria: i forni neolitici di Portonovo*. (Museo e istituto fiorentino di preistoria ‘Paolo Graziosi,’ 2019).
14. Paola Catalano; Stefania Di Giannantonio. *anthropological study of the human remains from the archaeological excavation of portonovo-fosso fontanaccia;* published in Origini n. Xxxv/2013. (gangemi editore, 2017).
15. ROBERTA LELLI. *BONE COLLAGEN CARBON (13C) AND NITROGEN (15N) STABLE ISOTOPE ANALYSIS OF HUMAN AND FAUNAL SAMPLES FROM PORTONOVO;PUBLISHED IN ORIGINI N*. (GANGEMI EDITORE, 2017).
16. Lelli, R. *et al.* Examining dietary variability of the earliest farmers of South-Eastern Italy. *Am. J. Phys. Anthropol.* **149**, 380–390 (2012).
17. Pessina, A. & Tiné, V. *Archeologia del Neolitico: l’Italia tra VI e IV millennio a.C.* (Carocci, 2009).
18. *Studi sul neolitico del Tavoliere della Puglia: indagine territoriale in un’area-campione*. (BAR, 1983).
19. Tafuri, M. A. *et al. Early Farmers*. (British Academy, 2014).
20. Tafuri, M. A. *et al.* Life and Death in Neolithic Southeastern Italy: The Strontium Isotopic Evidence. *International Journal of Osteoarchaeology* **26**, 1045–1057 (2016).
21. Fiorentino, G. *et al.* Climate changes and human–environment interactions in the Apulia region of southeastern Italy during the Neolithic period. *The Holocene* **23**, 1297–1316 (2013).
22. Mariotti, V., Muntoni, I. M. & Belcastro, M. G. New insights into the funerary rituals of the Neolithic site of Passo di Corvo (Apulia, Italy): The study of the human remains. *Journal of Archaeological Science: Reports* **34**, 102643 (2020).

23. Manfredini, A. & Cassano, S. M. *Masseria Candelaro: vita quotidiana e mondo ideologico in un villaggio neolitico sul Tavoliere*. (Grenzi, 2004).

24. Barbaro, C. C., Muntoni, I. M. & Marconi, N. Space, structures and society in the ditched village of Masseria Candelaro, on the Apulian Tavoliere, Italy.

25. Venturo, D. La necropoli neolitica di Contrada Galliano, Palagiano (Taranto). *La necropoli neolitica di Contrada Galliano, Palagiano (Taranto)* 297–305 (2017).

26. Radina, F. Società neolitiche del sud-est italiano tra VI e V millennio a.C. : simboli e modelli di circolazione mediterranea nella documentazione funeraria. *Società neolitiche del sud-est italiano tra VI e V millennio a.C. : simboli e modelli di circolazione mediterranea nella documentazione funeraria* 109–124 (2020) doi:10.32097/1119.

27. Oione, D., Muntoni, I. M., Quero, T., Modesto, R. & D'Ardes, A. La necropoli di tombe a grotticella del Neolitico finale di Cava Ripatetta (Lucera – FG). Risultati preliminari. in *Ipogei. La vita, la morte, i cultu in mondi sotterranei.* vol. XV in press (2020).

28. Mazzieri, P., Cavazzuti, C. & Quero, T. Nuove evidenze funerarie dell'età del Rame e del Bronzo antico dalle Marche: Fermo-San Claudio. in *Sepolture tra età del Rame r Bronzo antico: nuove scoperte* 46–50.

29. *Atti della XXXVIII Riunione scientifica: preistoria e protostoria delle Marche, Portonovo, Abbadia di fiastra, 1-5 ottobre 2003*. (Istituto Italiano di Preistoria e Protostoria, 2005).

30. Quero, T. *et al. Ipogei funerari eneolitici nell'alto Tavoliere e nel Subappennino Daunio. I cadi di Torre de Rubeis - Troia e Masseria D'Amendola - Deliceto*. (2020).

31. Barich, B. E., Bonadonna, F. P., Borgognoni, S. & Parenti, R. Trovamenti eneolitici presso Tarquinia. in *Origini - Preistoria e Protostoria delle civiltà antiche* vol. 2 173–246.

32. Passariello, I. *et al.* Contribution of Radiocarbon Dating to the Chronology of Eneolithic in Campania (Italy). *Geochronometria* **35**, 25–33 (2010).

33. Fugazzola Delpino, M. A., Tagliacozzo, A. & Vanzetti, A. Una comunità della cultura di Laterza nella pianura campana: l'area 'Forum' di Gricignano-US Navy (CE). in *Atti XXXV Riunione Scientifica dell'IIPP in memoria di L. Bernabò Brea.* 199–214.

34. Sperduti, A. *et al.* Tooth grooves, occlusal striations, dental calculus, and evidence for fiber processing in an Italian eneolithic/bronze age cemetery. *Am J Phys Anthropol* **167**, 234–243 (2018).

35. Ottoni, C. *et al.* Tracking the transition to agriculture in Southern Europe through ancient DNA analysis of dental calculus. *Proc Natl Acad Sci USA* **118**, e2102116118 (2021).

36. Gloor, G. B., Macklaim, J. M., Pawlowsky-Glahn, V. & Egozcue, J. J. Microbiome Datasets Are Compositional: And This Is Not Optional. *Front Microbiol* **8**, 2224 (2017).

37. Aitchison, J., Barceló-Vidal, C., Martín-Fernández, J. A. & Pawlowsky-Glahn, V. Logratio analysis and compositional distance. *Mathematical Geology* 271–275 (2000).

38. Wibowo, M. C. *et al.* Reconstruction of ancient microbial genomes from the human gut. *Nature* **594**, 234–239 (2021).

39. Kunin, V., Sorek, R. & Hugenholtz, P. Evolutionary conservation of sequence and secondary structures in CRISPR repeats. *Genome Biol* **8**, R61 (2007).

40. Barrangou, R. *et al.* CRISPR provides acquired resistance against viruses in prokaryotes. *Science* **315**, 1709–1712 (2007).

41. Guo, Y. *et al.* Antitoxin HigA inhibits virulence gene *mvfR* expression in *Pseudomonas aeruginosa*. *Environ Microbiol* **21**, 2707–2723 (2019).

42. Hahn, M. W., Moore, E. R. & Höfle, M. G. Bacterial filament formation, a defense mechanism against flagellate grazing, is growth rate controlled in bacteria of different phyla. *Appl Environ Microbiol* **65**, 25–35 (1999).

43. Komano, T., Utsumi, R. & Kawamukai, M. Functional analysis of the fic gene involved in regulation of cell division. *Res Microbiol* **142**, 269–277 (1991).

44. Klein, J. R., Henrich, B. & Plapp, R. Molecular analysis and nucleotide sequence of the envCD operon of Escherichia coli. *Mol Gen Genet* **230**, 230–240 (1991).

45. Kieboom, J. & de Bont, J. Identification and molecular characterization of an efflux system involved in Pseudomonas putida S12 multidrug resistance. *Microbiology (Reading)* **147**, 43–51 (2001).

46. Kuhn, H. M., Meier-Dieter, U. & Mayer, H. ECA, the enterobacterial common antigen. *FEMS Microbiol Rev* **4**, 195–222 (1988).

47. Rahim, R., Burrows, L. L., Monteiro, M. A., Perry, M. B. & Lam, J. S. Involvement of the rml locus in core oligosaccharide and O polysaccharide assembly in Pseudomonas aeruginosa. *Microbiology (Reading)* **146 (Pt 11)**, 2803–2814 (2000).

48. Henry, A. G., Brooks, A. S. & Piperno, D. R. Microfossils in calculus demonstrate consumption of plants and cooked foods in Neanderthal diets (Shanidar III, Iraq; Spy I and II, Belgium). *Proceedings of the National Academy of Sciences of the United States of America* **108**, 486–491 (2011).

49. Ma, Z., Perry, L., Li, Q. & Yang, X. Morphological changes in starch grains after dehusking and grinding with stone tools. *Sci Rep* **9**, 2355 (2019).

50. Li, W., Pagán-Jiménez, J. R., Tsoraki, C., Yao, L. & Van Gijn, A. Influence of grinding on the preservation of starch grains from rice. *Archaeometry* **62**, 157–171 (2020).

51. Haslam, M. The decomposition of starch grains in soils: implications for archaeological residue analyses. *Journal of Archaeological Science* **31**, 1715–1734 (2004).

52. Stoddard, F. L. & Sarker, R. Characterization of starch in Aegilops species. *Cereal Chemistry* **77**, 445–447 (2000).

53. Yang, X. & Perry, L. Identification of ancient starch grains from the tribe Triticeae in the North China Plain. *Journal of Archaeological Science* **40**, 3170–3177 (2013).

54. Howard, T. *et al.* Identification of a major QTL controlling the content of B-type starch granules in Aegilops. *Journal of Experimental Botany* **62**, 2217–2228 (2011).

55. Ball, T. B., Gardner, J. S. & Anderson, N. Identifying inflorescence phytoliths from selected species of wheat (*Triticum monococcum, T. dicoccon, T. dicoccoides,* and *T. aestivum*) and barley (*Hordeum vulgare* and *H. spontaneum*) (Gramineae). *Am. J. Bot.* **86**, 1615–1623 (1999).

56. Alikhan, N.-F., Petty, N. K., Ben Zakour, N. L. & Beatson, S. A. BLAST Ring Image Generator (BRIG): simple prokaryote genome comparisons. *BMC Genomics* **12**, 402 (2011).
