# Supplementary material for: Ancient oral microbiomes support gradual Neolithic dietary shifts towards agriculture
Source: Nat Commun. 2022 Nov 22;13:6927. doi: 10.1038/s41467-022-34416-0 (PMC9681849; doi:10.1038/s41467-022-34416-0)
Supplement: Supplementary file 2 — Description of Additional Supplementary Files [file 41467_2022_34416_MOESM2_ESM.pdf]

Description of Additional Supplementary Information

File name: Supplementary Data 1
Description: Dataset metadata. Archaeological, anthropological, cultural and molecular information about the samples analysed in the present study. The "DNA" and "Microscopic analysis" columns indicate if the sample was for metagenomic analysis or for the identification of micro-debris embedded within dental calculus. The "Source tracker (% oral)" column report the percentage of oral microbiome detected through Sourcetracker analysis. We reported, when available, direct pathological information about each sample or a general evaluation of the oral health status by site as performed by the analysis of skeletal remains (see Supplementary information for details about each site). Direct radiocarbon date is reported when available. Dates reported in bold refers to directly dated individuals, while dates in italic refer to associated remains. If no direct C14 analysis was performed, the samples were dated on the basis of relative chronology.

File name: Supplementary Data 2
Description: Sequencing outputs. Number of reads (both raw and cleaned) for each sample analysed. The proportion of bacterial, archaea and eukaryote reads are reported.

File name: Supplementary Data 3
Description: Microbiome validation. Ancient and modern metagenomic samples from literature used as a reference of human microbiome variability to validate the microbiome profile obtained from the ancient samples analysed in the present study.

File name: Supplementary Data 4
Description: Ancient DNA damage. Post-mortem damage and -Δ % for the most abundant microbial species identified in ancient samples. After alignment to the reference genome, species were filtered based on their mapping quality and post-mortem damage (MAPQ=30 and PMD=1, respectively)

File name: Supplementary Data 5
Description: PCoA Statistical analyses. A) PERMANOVA and ANOSIM results performed on the PCoA analyses are reported. b) Pairwise ANOSIM performed between pairs of clusters on the PCoA analysis.

File name: Supplementary Data 6
Description: DESeq2 results. Results obtained from DESeq2 analysis are reported, each column reports the adjusted p-value for each cluster comparison; red cells indicate adjusted p-value <0.05.

File name: Supplementary Data 7
Description: MaAsLin results. Results obtained from MaAsLin are reported: red and green cells in Coefficient column indicate if the association had positive or negative values, respectively.

File name: Supplementary Data 8
Description: Micro-Debris detected in dental calculus. Report of the micro-debris identified within Palaeolithic, Neolithic and Copper Age dental calculus samples.

File name: Supplementary Data 9
Description: Genome assembly. Results from *de novo* reconstruction of *Olsenella sp. oral taxon 807* in six ancient dental calculus samples. Several information is reported about genome completeness, contamination level, coverage, N50 values, mean contig length and size of the largest contig.

File name: Supplementary Data 10
Description: *Olsenella sp. oral taxon 807* proteins. Protein families resulted from Protein Family Sorter tool within PATRIC workspace. The number of proteins in a particular family is reported.

File name: Supplementary Data 11
Description: Species abundance. Relative abundance of the species identified by the metagenomic analysis.
