# Supplementary material for: Ancient oral microbiomes support gradual Neolithic dietary shifts towards agriculture
Source: Nat Commun. 2022 Nov 22;13:6927. doi: 10.1038/s41467-022-34416-0 (PMC9681849; doi:10.1038/s41467-022-34416-0)
Supplement: Supplementary file 14 — Reporting Summary [file 41467_2022_34416_MOESM14_ESM.pdf]

nature portfolio

Corresponding author(s): DBPR NCOMMS-22-08281

Last updated by author(s): Oct 15, 2022

Reporting Summary

Nature Portfolio wishes to improve the reproducibility of the work that we publish. This form provides structure for consistency and transparency in reporting. For further information on Nature Portfolio policies, see our Editorial Policies and the Editorial Policy Checklist.

Statistics

For all statistical analyses, confirm that the following items are present in the figure legend, table legend, main text, or Methods section.

n/a	Confirmed	
☐	☒	The exact sample size (*n*) for each experimental group/condition, given as a discrete number and unit of measurement
☐	☒	A statement on whether measurements were taken from distinct samples or whether the same sample was measured repeatedly
☐	☒	The statistical test(s) used AND whether they are one- or two-sided<br>*Only common tests should be described solely by name; describe more complex techniques in the Methods section.*
☐	☒	A description of all covariates tested
☐	☒	A description of any assumptions or corrections, such as tests of normality and adjustment for multiple comparisons
☐	☒	A full description of the statistical parameters including central tendency (e.g. means) or other basic estimates (e.g. regression coefficient) AND variation (e.g. standard deviation) or associated estimates of uncertainty (e.g. confidence intervals)
☐	☒	For null hypothesis testing, the test statistic (e.g. *F*, *t*, *r*) with confidence intervals, effect sizes, degrees of freedom and *P* value noted<br>*Give P values as exact values whenever suitable.*
☒	☐	For Bayesian analysis, information on the choice of priors and Markov chain Monte Carlo settings
☒	☐	For hierarchical and complex designs, identification of the appropriate level for tests and full reporting of outcomes
☒	☐	Estimates of effect sizes (e.g. Cohen's *d*, Pearson's *r*), indicating how they were calculated

Our web collection on statistics for biologists contains articles on many of the points above.

Software and code

Policy information about availability of computer code

Data collection	Illumina sequence data were processed using the following programs: AdapterRemoval (v2.2.0),Prinseq (v.0.19.1), Kraken2 (v.2.0.8-beta), Bracken (v.2.5), Dustmasker (v.1.0.0), Burrows-Wheeler Alignment (BWA v.0.7.15), PMDtools (v.0.60), OxCal (v.4.4). These programs are publicly available
Data analysis	Data analyses were performed using several packages from R software (v.3.6.3): microViz (v.0.9.0), cluster (v.2.1.0), phyloseq (v.1.30.0), NetCoMi(v.1.0.2), k-nearest neighbour (k-nn) algorithm is implemented in NetCoMi package, radiant (v.1.4.0), vcd (v. 1.4-9), DESeq2 (v.1.26.0), ggplot2 (v.3.3.5).<br>Other programs were used for microbiome data analysis: HUMAnN 2.0, LEfSe (v.1.0), MaAsLin v.1.0<br>Metagenomes assembly and analysis were performed using: SPAdes (v.3.15.3), SAMtools (v.1.9), Bowtie2 (v.2.3.5.1), MetaBAT 2 (v.2.12.1), CheckM (v.1.1.6), BLAST Ring Image Generator (BRIG) (v.0.95), PATRIC (v.3.6.12), RAxML (v.8.2.11)

For manuscripts utilizing custom algorithms or software that are central to the research but not yet described in published literature, software must be made available to editors and reviewers. We strongly encourage code deposition in a community repository (e.g. GitHub). See the Nature Portfolio guidelines for submitting code & software for further information.

Data

Policy information about availability of data

All manuscripts must include a data availability statement. This statement should provide the following information, where applicable:

- Accession codes, unique identifiers, or web links for publicly available datasets
- A description of any restrictions on data availability
- For clinical datasets or third party data, please ensure that the statement adheres to our policy

All the sequence data generated for this study have been deposited BioProject Database with the ID: PRJNA791766
Code is available at https://github.com/AndreaQ7/HuME/
Human Oral Microbiome Database is available at https://www.homd.org/
NCBI Reference Sequence (RefSeq) database is available at https://www.ncbi.nlm.gov/refseq/

Human research participants

Policy information about studies involving human research participants and Sex and Gender in Research.

Reporting on sex and gender	NA
Population characteristics	NA
Recruitment	NA
Ethics oversight	NA

Note that full information on the approval of the study protocol must also be provided in the manuscript.

Field-specific reporting

Please select the one below that is the best fit for your research. If you are not sure, read the appropriate sections before making your selection.

☐ Life sciences ☐ Behavioural & social sciences ☒ Ecological, evolutionary & environmental sciences

For a reference copy of the document with all sections, see nature.com/documents/nr-reporting-summary-flat.pdf

Ecological, evolutionary & environmental sciences study design

All studies must disclose on these points even when the disclosure is negative.

Study description	This study includes the analysis of 79 ancient dental calculus from hunter-gatherer Paleolithic (31,000 BC), Neolithic farmers (6,200-4,000) and Copper Age (3,500-2,000), combining de novo metagenomic sequencing (76 samples) with microscopic analysis (27 samples). Microbiome profiles were obtained thought Kraken2 and Bracken softwares, and analysed for their biodiversity using several R software packages.
Research sample	Research samples are composed by three main cultural groups: Hunter-gatherer from Paleolithic, early farmers' communities from Neolithic and farmers' samples from Copper Age. Further sub-division based on specific cultural background (archaeologically-defined) were reported on SI and Supp. Table 1. We collected all the available archaeological, cultural and anthropological information for each sample and site considered (see Supp. Table 1). Samples were analysed for their oral microbial biodiversity with an unsupervised approach, performing cluster analysis to highlight possible groups within our dataset. Then, we associate the identified clusters with the available metadata, in order to identify the main variable at the basis of cluster division.
Sampling strategy	In order to minimize the effect of geography on the microbiome composition, we decide to collect samples that belong to a restricted geographic area across a long period of time. Moreover, samples that belong to multiple coeval archaeological sites were collected in order to discriminate the possible site-effect on the overall trend associable to each time period considered in this study.
Data collection	Depending on samples location, different local authorities and researchers were present during the collection. Details about their role during the collection of samples belonging to different site and periods are reported in the Author Contributions section of the article. A.Quagliariello and A.Modi were involved in dental calculus collection using 2mL sterile tubes. Archaeological and Anthropological data were transferred through Excel files from the local Archaeological autorities to A.Quagliariello. Samples were extracted and sequenced at the dedicated aDNA clean laboratory at the Department of Biology of the University of Florence (Italy), using Illumina NovaSeq 6000 platform in paried-end mode with 2x51+8+8 cycles.
Timing and spatial scale	Samples were analyzed over the period January 2020 - February 2021. Detailed information about the arcaheological samples analyzed are provided in Supp.Table 1, SI and Fig. 1.

Data exclusions	We excluded samples only if the samples do not meet the quality criteria, either by having low level of endogenous microbial DNA or by showing high level of environmental contamination estimates.
Reproducibility	We took multiple individuals from each archaeological site, if available, to support the representativeness of their metagenomic profiles. Due to the limited amount and the precious nature of the archaeological biological materials, repeated genome data collection on the same individual was not performed. As required by the field standards, several bioinformatic analyses were applied to the sequencing data of each sample in order to authenticate ancient microbiome data and exclude possible environmental contamination. Each samples required to pass a quality control to ensure the presence of authentic endogenous ancient DNA and no environmental contamination. Repeated genome data collection on the same individual is not considered as necessary nor useful in the field standard and therefore was not performed.
Randomization	Randomization was not applicable because this study is observational and did not include any case/control comparison or treatment.
Blinding	There was no experimental treatment of samples involved in this study that requires blinding. Data analysis was performed based on the analysis groups that were defined by external information such as archaeological context and date.

Did the study involve field work? ☒ Yes ☐ No

Field work, collection and transport

Field conditions	Samples have been previously excavated, no new excavation were performed. Samples were conserved in different museums and archaeological archive of the Italian Heritage Ministry.
Location	Samples were stored principally in three different locations: at the archaeological archive of Italian Heritage Ministry in Foggia (41.45 lat, 15.55 long), at the Museums and at the archaeological archive of Italian Heritage Ministry of Ancona (43.5 lat, 13.5 long), and at different Museums in the city of Rome (41.89 lat, 12.51 long).
Access & import/export	Samples were collected in accordance with local authorities and with the cooperation of the local archaeologists. Every samples collected received a signed authorization to the treatment and analysis based on written agreement (received before the start of the project).
Disturbance	N/A

Reporting for specific materials, systems and methods

We require information from authors about some types of materials, experimental systems and methods used in many studies. Here, indicate whether each material, system or method listed is relevant to your study. If you are not sure if a list item applies to your research, read the appropriate section before selecting a response.

Materials & experimental systems

n/a	Involved in the study
☒	☐ Antibodies
☒	☐ Eukaryotic cell lines
☐	☒ Palaeontology and archaeology
☒	☐ Animals and other organisms
☒	☐ Clinical data
☒	☐ Dual use research of concern

Methods

n/a	Involved in the study
☒	☐ ChIP-seq
☒	☐ Flow cytometry
☒	☐ MRI-based neuroimaging

Palaeontology and Archaeology

Specimen provenance	Various archaeological sites in Italy; see Supp.Table 1 and the SI for a comprehensive list of archaeological sites, locations, and archaeological/anthrpological information.
Specimen deposition	Contact information for each specimen is provided in the SI
Dating methods	Radiocarbon dates both calibrated (2 sigma) and uncalibrated are provided at Supp. Table 1.

☒ Tick this box to confirm that the raw and calibrated dates are available in the paper or in Supplementary Information.

Ethics oversight	Study consists of the analysis of previously excavated human archaeological remains, and included no new excavation effort nor study of live human or animal subjects excavation. Partners providing specimens are coauthors on the study. The access to the

remains were approved by the Italian Heritage Ministry based on the written agreements. Specifically, archaeological samples were collected at the “Soprintendenza Archeologia, Belle Arti e Paesaggio per la Città metropolitana di Bari” in Bari (Italy), at the “Soprintendenza Archeologia, Belle Arti e Paesaggio per le Province di Barletta- Andria -Trani e Foggia” in Foggia (Italy), at the “Soprintendenza Archeologia, Belle Arti e Paesaggio delle Marche” in Ancona (Italy), at the “Museo delle origini” at the University “La Sapienza” in Rome (Italy), and at the museum “Museo delle Civiltà” in Rome (Italy). Each local authority listed above approved the sample collection by written agreement.

Note that full information on the approval of the study protocol must also be provided in the manuscript.
